# Orthodontic Treatment in Patients With Epidermolysis Bullosa (EB)—Clinical Practice Guidelines (CPG)

**DOI:** 10.1111/scd.70084

**Published:** 2025-09-03

**Authors:** Sebastián Véliz, María Teresa Abeleira, María Concepción Serrano, Carole Charavet, Kirsten FitzGerald, Francisca Hormazábal, Hinrich Huber, María Korolenkova, Suhaym Mubeen, Sanchit Paul, Gabriela Scagnet, Lorena Sepúlveda, Elena Syomkina, Svetlana Tekucheva, An Verdonck, Anne W. Lucky, Cristina Has, Gudrun Salamon, Susanne Krämer

**Affiliations:** ^1^ Facultad De Odontología Universidad De Chile Santiago Chile; ^2^ Facultad De Medicina y Odontoloxia Universidad De Santiago de Compostela Santiago de Compostela Spain; ^3^ Sigmund Freud Universität Vienna Austria; ^4^ Private Practice Valencia Spain; ^5^ Département Orthopédie Dento‐Faciale (ODF) Université Côte d'Azur Laboratoire MICORALIS Nice France; ^6^ CHU De Nice, Institut de Médecine Bucco‐Dentaire Unité Soins des Enfants (ODF) Nice France; ^7^ Children's Health Ireland Crumlin Ireland; ^8^ Central Research Institute of Dentistry and Maxillofacial Surgery Moscow Russia; ^9^ Great Ormond Street Hospital London UK; ^10^ Tooth Tales: Nurturing Healthy Smiles Greater Noida India; ^11^ Hospital De Odontopediatría Martín Gobierno Ciudad de Buenos Aires Universidad De Buenos Aires Buenos Aires Argentina; ^12^ KULeuven Department of Oral Health Sciences| Orthodontics Belgium University Hospitals Leuven Leuven Belgium; ^13^ Cincinnati Children's Epidermolysis Bullosa Center Cincinnati Children's Hospital Cincinnati Ohio USA; ^14^ Department of Dermatology Medical Faculty and Medical Centre University of Freiburg Freiburg im Breisgau Germany

## Abstract

**Background:**

Epidermolysis bullosa (EB) is a rare genetic condition characterized by skin and mucosal fragility. The clinical phenotype is highly variable. Severe types and subtypes, such as junctional EB (JEB), kindler EB (KEB), and recessive dystrophic EB (RDEB), are considered to present a high risk of oral health problems, including malocclusions. Despite this, the literature on orthodontic treatment in patients with EB is scarce and is limited to a few case reports.

**Objective:**

To provide the users with information on the current best practices for orthodontic and dentofacial orthopedic diagnosis and treatment for patients with EB.

**Methods:**

Current information regarding orthodontic treatment in patients with EB was identified based on a systematic literature review. A panel of experts was invited to provide additional information based on their experience through an open‐ended form. Later, a Delphi study was performed over two rounds with a consensus threshold at 75%. Members of the medical team and patient representatives revised the final document.

**Results:**

The panel (n = 12) agreed on a total of 15 recommendations, divided into three categories: general information on EB and orthodontics; orthodontic diagnosis and orthodontic treatment. A fourth category on perspectives was developed based on the feedback provided by non‐dental members of the medical team (*n* = 4) and patients (*n* = 2).

**Conclusions:**

Orthodontic treatment guidelines for patients living with EB are presented, including general aspects of EB, orthodontic diagnosis, and orthodontic treatment.

## 1 Introduction

Inherited epidermolysis bullosa (EB) is a rare disease characterized by skin and mucosal fragility. It is caused by genetic variants in structural proteins of the basement membrane zone, showing a variable phenotypic presentation with four major types and 36 subtypes [1]. These types are EB simplex (EBS), junctional EB (JEB), dystrophic EB (DEB), and kindler EB (KEB). According to their clinical presentation, each type has its subtypes, with recessive dystrophic EB (RDEB) being one of the most severe [1, 2].

The typical oral characteristic of all four types is skin and mucosal fragility, causing oral lesions, including bullae, ulcers, erosions, and vesicles develop under traction/shear forces that in the non‐EB population generally would not cause any harm [3]. Other characteristics might depend on the subtype. In RDEB, oral strictures such as microstomia, ankyloglossia, and vestibule obliteration can present, as well as a high caries index, and absence of tongue papillae and palatal rugae. In patients with JEB, syndromic amelogenesis imperfecta (AI) can be found as generalized hypoplastic or pitted hypoplastic enamel. JEB patients also get perioral granulation tissue, gingival hyperplasia, tooth retention, and crown resorption [4]. In KEB, early onset periodontal disease and pitting AI may also be present [3, 5].

The dental approach to patients with EB focuses on prevention and early access to appropriate care. Even though dental diseases can be challenging to control and several features can be progressive over time, this focus on prevention has led to better oral health in these patients' lives, modifying the natural history of the disease from early‐age edentulism to patients with their teeth until adulthood [3]. Improving their oral health and quality of life has required the participation of more dental specialties, one of them being orthodontics, as malocclusions are a common feature in patients with EB, such as RDEB [6].

Multiple cases of orthodontic treatment in EB have been reported as new technologies in this field emerge, such as aligners and mini‐implants [7, 8]. Even so, access to these treatments remains scarce due to barriers, such as economic burden, lack of trained professionals in rare diseases, or long‐distance travel [3, 9]. The present Clinical Practice Guidelines (CPG) on orthodontic treatment in patients with EB seek to fight access barriers and improve the quality of treatments for people living with EB. It has been developed using a standard methodology based on a systematic review of the scientific evidence and assessed by an international panel of experts in EB, including orthodontists, pediatric dentists, special care dentists, speech therapists, and dermatologists, using a consensus strategy with Delphi methodology.

## 2 Scope and Purpose

### 2.1 Aim

To provide the users with information on the current best practices (BPs) for orthodontic and dentofacial orthopedic (DO) diagnosis and treatment for patients with EB.

### 2.2 Users

Specialist in orthodontics, pediatric dentistry, and special care dentistry. This CPG will provide information and recommendations for dentists and specialists in the field of orthodontics with a special approach for patients living with EB. This is not a guideline for dentists to learn how to perform orthodontic treatment. Patients or other healthcare providers, especially dermatologists and primary care providers, can benefit from its information, but it will not enable them to perform this type of treatment.

### 2.3 Target Group

These guidelines can be applied to all patients diagnosed with EB who require or are under orthodontic or DO treatment, with no gender or age limitation.

## 3 Questions

The selected questions were defined based on the literature analysis and addressed by a group of experts using consensus Delphi methodology:

1. General information about DO and orthodontic treatment in EB.
  1.1 Is orthodontic and/or DO treatment possible in patients living with EB?
  1.2 Is orthodontic and/or DO treatment the same in complexity for all patients with EB?
  1.3 At what age should patients diagnosed with EB be referred to an orthodontist for their initial orthodontic evaluation?
2. Orthodontic and DO diagnosis in EB.
  2.1 What main characteristics can be observed in patients with EB?
  2.2 What types of malocclusions or orthodontic problems are most common among EB patients?
  2.3 Which methods are necessary to achieve a successful dentofacial orthodontic diagnosis in patients with EB?
3. Orthodontic and DO treatment in EB.
  3.1 What are the benefits of orthodontic treatment in patients with EB?
  3.2 What are the multidisciplinary areas that patients with EB may require prior to, during, or subsequent to receiving orthodontic treatment?
  3.3 Which considerations should be taken into account when planning orthodontic treatment for patients with EB?
  3.4 Which adjustments can be implemented to adapt orthodontic techniques for patients with EB?
  3.5 Is orthodontic retention necessary for individuals with EB?
  3.6 What are the complications associated with orthodontic treatment in individuals diagnosed with EB?
  3.7 What considerations are necessary for maintaining oral hygiene in patients with EB who are undergoing orthodontic treatment?
  3.8 What are the barriers patients with EB face when it comes to orthodontic treatment?
  3.9 What adjustments for orthodontic treatment are needed by patients with EB subtypes with low and moderate risk of oral and dental manifestations and complications?
4. Other groups' perspectives on this guide
  4.1 What are the perspectives of patients and other members of the medical team regarding this CPG?

## 4 Systematic Literature Searching

### 4.1 Literature Sources

The literature search ranged from 1994 to August 2024 (the last 30 years). Consulted sources included the electronic databases PUBMED (MeSH, tiab), EMBASE (Emtree), The Cochrane Library (DARE), the Cochrane Controlled Trials Register (CENTRAL), and SCIELO. Hand‐searching, including journals, conferences, and other sources of guidelines, was assessed. Unpublished documents that meet the selection criteria, such as dissertations, conference and technical reports, were also included.

### 4.2 Selection Criteria of the Articles

Articles that mention relevant and specific information about orthodontic and/or DO diagnosis and/or treatment in patients with EB, published between 1994 and August 2024 in any language, were included. All articles were screened, with a first stage based on the title and the abstract and a second stage based on the full article. The criteria to reject articles at both stages were:(a) The article is unrelated to inherited EB. (b) The article describes dental treatment in patients with EB but does not include DO or orthodontic information, such as diagnosis or treatment. (c) The cohort has already been included in a different article. (d) Literature review, which does not provide new information.

### 4.3 Search Strategy

The search strategy used to identify proper articles was developed for MEDLINE and adapted to each database, using a compilation of controlled vocabulary and free text based on the following terms.

#1 Epidermolysis Bullosa: ti, ab, kw

#2 (Acantholysis Bullosa): ti, ab, kw

#3 MeSH descriptor Epidermolysis Bullosa exploded all trees

#4 MeSH descriptor Orthodontics exploded all trees

#5 MeSH descriptor Malocclusion exploded all trees

#6 (malocclusion): ti, ab, kw

#7 (crossbite): ti, ab, kw

#8 (orthodontic): ti, ab, kw

#9 (oral functions): ti, ab, kw

#10 #1 OR (#2 OR (#3))

#11 #4 OR (#5)

#12 #6 OR (#7 OR (#8 (#9)))

#13 #11 OR #12

#14 #10 AND #13

(“Epidermolysis Bullosa”[Text Word] OR “acantholysis bullosa”[Text Word] OR “Epidermolysis Bullosa”[MeSH]) AND (“orthodontics”[MeSH Terms] OR “Malocclusion”[MeSH] OR “malocclusion”[Text Word] OR “crossbite”[Text Word] OR “orthodontic”[Text Word] OR “oral functions”[Text Word]).

### 4.4 Method Used for the Formulation of the Recommendation and Report

The SIGN Guidelines were used to formulate the following recommendations and the AGREE guidelines for reporting CPG [10, 11].

### 4.5 Clinical Experts and Patient Representatives

The information selected was complemented and assessed by a group of professionals with experience in EB who had previously performed any orthodontic or DO treatment. They were recruited through the IADH (International Association for Disability and Oral Health) research network (register at: http://iadh.org/research/) and Debra International networks. Inclusion criteria were those centers/professionals who reported performing orthodontic treatment on people with rare diseases, specifically EB. A total of 12 professionals participated, including orthodontists, pediatric dentists, and special care dentists. The information was assessed using a Delphi approach with a threshold of agreement set at 75%. After two rounds, total agreement was obtained. The perspectives of four medical team members (dermatologist, psychologist, and speech therapist) and two patients regarding this CPG were also included. External revision was performed by an orthodontist with experience in EB who did not participate in the panel. There were a total of 12 countries participating in this project. (Tables S1, S2, and S3).

## 5 Guidelines Implementation

### 5.1 Implementation Barriers

Even though this CPG is intended to improve access to orthodontic treatment for patients living with EB, the implementation of these CPG can face barriers according to the local context, including:

- Limited access to dental treatment.
- Lack of access to a multidisciplinary team that includes a DO or orthodontic treatment.
- Lack of proper training in orthodontics or DO.
- Economic limitations to provide orthodontic treatment.

### 5.2 Implementation Strategy

Each team will be responsible for the implementation of this CPG. Mechanisms should be considered to incorporate orthodontic treatment for patients with EB, in case the patient needs it, and to review the provided care, considering local adaptation.

### 5.3 Cost Implications

Cost analysis should be performed according to the local context. Orthodontic treatment should consider professional hours, appropriately trained personnel, dental equipment, orthodontic material (including brackets, wires, pliers, etc.), and the orthodontic laboratory.

### 5.4 Further Areas of Research

- To evaluate orthodontic treatment needs in EB.
- To evaluate different orthodontic treatment strategies, their challenges, and success rates in EB.
- To evaluate the impact of orthodontic or DO treatment on oral functioning, including deglutition, masticatory function, and parafunctional habits in people with EB.

### 5.5 Guideline Updating Procedure

The guideline will be updated every 5 years after this guideline publication. The team in charge of this update will be led by Dr Susanne Krämer and Dr. Sebastián Véliz, who will be recruiting professionals working in this subject by then. Authors can be contacted for monitoring these recommendations.

## 6 Results

### 6.1 Literature Search

A total of 14 articles were found: one cross‐sectional study, five case–control studies (Table 1), and eight case reports (Table 2). Review articles and articles on other topics were excluded.

**TABLE 1:** Summary of evidence‐based studies regarding orthodontic diagnosis reported in patients with EB.

Authors	Study type	Country	Patient characteristics	Intervention	Outcomes	Conclusions	Limitations for this CPG
Shah, 2002 [28]	Case‐control	UK	42 RDEB patients	Lateral cephalometric radiograph assessment compared with published longitudinal data.	Maxillary length, mandibular length, middle facial height, lower facial height, and distance from the lower lip to the esthetic plane were significantly reduced in RDEB compared to the control group. Saddle and nasolabial angles were significantly larger in the RDEB group.	The combined effects of malnutrition and scarring cause a marked inhibition of facial growth in RDEB patients, contributing significantly to marked dento‐alveolar disproportion and consequently to dental crowding.	Only RDEB
De Benedittis, 2004 [25]	Case‐control	Italy	6 RDEB patients	Oral hard and soft tissue involvement compared to 36 children	Reduced mouth opening, intraoral involvement with blistering and erosions, absence of enamel hypoplasia, high dental caries index, poor oral hygiene, 4/6 patients showed crowded teeth, and 2/6 showed crossbite.	Dental prophylaxis should commence as soon as tooth eruption begins, being particularly important because of high caries index, tooth malpositions, and the precocious onset of tissue destinations, mostly microstomia.	Only RDEB, a low number of participants.
Stellingsma, 2011 [22]	Cross‐sectional	Netherlands	22 EB patients (10 RDEB, 8 JEB, 3 DDEB, and 1 EBS)	Assessment of the mobility of the mandible, lips, and tongue, and a questionnaire about mandibular function impairment.	Mobility of the mandible, tongue, and lips was less in RDEB. Mandibular function was more severely impaired in RDEB. Oral hygiene was hindered in most patients with EB.	Restriction in mobility of the mouth, tongue, and lips is frequent in EB patients, being more severe in RDEB.	No correlation between restrictions in mobility and malocclusions was assessed.
Poberezhnaya, 2021 [23]	Case‐control	Russia	50 EB (45 RDEB, 3 JEB, 1 KEB, and 1 EBS) patients	Measure mouth opening distance, inter‐commissure distance and tongue movement.	Mouth opening increases in DEB patients until mixed dentition, followed by a drastic decrease after 12 years old. Similar data about inter‐commissure distance and tongue mobility was obtained.	It is necessary to develop strategies to reduce microstomia and ankyloglossia in patients with EB.	No correlation between restrictions in mobility and malocclusions was assessed
Korolenkova, 2021 [27]	Case‐control	Russia	22 EB patients	Panoramic radiographs were used to assess dental age and compared with healthy controls.	A higher percentage of EB patients (10/22) present a dental age higher than physical age, and only 5/22 showed a dental age corresponding to the physical age.	Possible dental age retardation in EB patients should be considered when undertaking dental extractions for orthodontic reasons.	Unclear effect in early extraction protocols
Korolenkova, 2022 [24]	Case‐control	Russia	27 DEB patients	Assessment of morphological and functional aspects of MOI and compared to healthy controls	EBOS values higher than 40 should be seen as unfavorable, as they are always associated with severe functional restrictions	The EBOS may be useful in assessing the impact of oral mucosa on disease prognosis.	No correlation between restrictions in mobility and malocclusions was assessed.

**TABLE 2:** Summary of orthodontic treatments reported in patients with EB.

Authors	Age, EB type, and variant	Orofacial features	Systemic features	Malocclusion diagnosis	Skeletal /Facial diagnosis	Functional anomalies	Preparation needed	Type of treatment (Time)	Adaptation required	Complications	Outcome	Retention
Goldschmied, 1999 [16]	10 years old. EB, non‐scarring type.	Impacted third molars,	Mild lesions and scarring in his body,	Angle Class II Division 1, narrow tapered mandibular arch	Skeletal Class II, mesofacial pattern	—	—	Fixed brackets, Begg treatment. 30 months	The author would have indicated using extraoral traction appliances, but considered them inappropriate.	—	Ideal.	Thermoplastic upper and lower retainers. After 2 years, the occlusion remained stable .
Pacheco, 2008 [15]	37 years old. RDEB	Trismus, oral lesions, limited mouth opening, poor oral hygiene, caries, hemorrhagic bullae in the mouth and larynx	Lesions in fingers and nails, body ulceration, and alopecia	Class II malocclusion with bilateral posterior crossbite and anterior crossbite in the area of teeth #26 and #27, severe crowding in the lower arch.	—	—	Preventive and periodontal treatment	Edgewise fixed braces combined with Hyrax.	The author would have indicated premolars’ extraction, but it was avoided due to EB, extracting #42	Oral ulcers due to braces, using orthodontic wax when necessary.	Ideal. Oral rehabilitation is needed after orthodontic treatment, including fixed cantilever and removable dentures.	Maxillary: Removable Hawley retainer. Mandibular: Removable spring retainer. Both were used for 6 months. After two years, the occlusion remained stable.
Portillo, 2014 [13]	8 years old. DEB	Microstomia, bullae, ulcers in the healing process, ankyloglossia, absence of tongue papillae, scar tissue, angular cheilitis, caries	Bullae	Mixed dentition, severe crowding, lack of space for permanent canines eruption	Normodivergent, decreased facial middle face height, convex profile, posterior position of the lower lip, and soft pogonion		Preventive and restorative treatment	Early teeth extraction.	A space maintainer was avoided due to EB.	—	—	—
	7 years old. DEB	Bullae, microstomia, Ankyloglossia, poor oral hygiene, caries	Bullae, difficulty walking	Mixed dentition, maxillary compression, and lack of space for permanent teeth.	—		Preventive and restorative treatment	Early teeth extraction		—	—	—
Véliz, 2020 [14]	9 to 12 years old. Intermediate RDEB. Heterozygous c.6527_6528insC and c.8329C>T in COL7A1	Vestibule obliteration, severe microstomia, mucosal fragility, absence of tongue papillae, and palatal rugae	Severe malnutrition, esophageal stenosis, mild scoliosis, chronic constipation, recurrent corneal ulcers, and fine fiber neuropathy.	Lack of space for upper permanent canine eruption	—	History of 2 esophageal dilatations	—	Early teeth extraction, #14 and #24		—	Ideal, 3 years after the upper arch is aligned	—
	4 to 18 years old. Severe RDEB. Homozygous c.6527_6528insC in COL7A1	Caries, absence of tongue papillae, vestibule obliteration, micostomia, oral bullae, and mucosal fragility	Progressive hand retraction, recurrent urinary infections, chronic constipation, fine fiber neuropathy, and vitamin D deficiency	Lack of space for upper permanent canine eruption	—	Dysphagia with a history of at least 10 esophageal dilatations	Extraction of #16, #26, #36 and #46 due to caries	Early teeth extraction, #14 and #24		—	Ideal, 8 years after the upper arch is aligned	—
	10 years old. Severe RDEB. Heterozygous c.6527_6528insC and c.7708delG in COL7A1	Severe microstomia (6 mm of interincisors opening), Upper and lower incisors aligned, ankyloglossia, and oral vestibule obliteration.	Severe malnutrition, failure to thrive, vitamin D deficiency, recurrent infections, chronic constipation, pseudosyndactyly, and musculoskeletal contractures	Severe crowding with protrusion of the upper central incisors, lack of space for the canines to erupt	—	Dysphagia with a history of at least 8 esophageal dilatations	Two years early, extraction of the deciduous upper lateral incisor and canines, and all deciduous lower incisors due to a traumatic ulcer	Early teeth extraction, #13 and #23	Permanent upper canines extraction instead of first premolars due to limited access and well‐alignment of lateral incisors and first premolars	—	Ideal, 1 month after the site healed uneventfully	—
Blanchet, 2021 [17]	7 to 14 years old. KEB. Homozygous g.80929_89169del in FERMT1	Teeth sensitivity, bleeding gums, poor oral hygiene, erosive lesions around the mouth, angular cheilitis, caries, enamel hypoplasia of all teeth, intraoral lesions, moderate microstomia,	Urethral stenosis, hydrocele, ingrown toenails, hyperhidrosis, and lesions on the skin of the hands	Short U‐shaped upper maxillar with a lack of space for the permanent canines to erupt, lack of space for the two left mandibular and the first right premolars to erupt	—	—	Preventive and restorative, including the extraction of a deciduous tooth before orthodontic treatment	First stage: Early teeth extraction of all four first premolars. Second stage: fixed braces to align the teeth and preserve the space for the canines. (3 years)		Oral ulcers due to braces, using orthodontic wax when necessary. Debonding braces due to enamel hypoplasia	Ideal checkups for a year after treatment with periodontal concerns.	—
Véliz, 2022 [7]	25 years old. Severe RDEB. Homozygous c.6527_6528insC in COL7A1	Facial erythema, erosions, milia, crust, scars, epithelial atrophy, oral ulcers and blisters, angular cheilitis, absence of palatal rugae and tongue papillae, tongue scalloping, ankyloglossia, vestibule obliteration, microstomia.	Hand retraction, recurrent urinary infections, osteopenia, and kidney disease	Class II div 2, Bilateral posterior crossbite, increased overbite, inferior crowding, severe retroclined lower incisors, upper midline shifted right.	Skeletal class II	Mouth breathing, atypical deglutition, dysphagia with history of at least 15 esophageal dilatations.	Oral surgery for microstomia and vestibule obliteration	Aligners	Flexible trays were developed to obtain impressions with additional silicone.	Oral ulcers during impression due to microstomia	—	—
Véliz, 2024 [18]	27 years‐old. Severe RDEB. Heterozygous c.6527_6528insC and c.7708delG in COL7A1	Facial erythema, epithelial atrophy, ulcers, erosions, scars, lip atrophy, granulation tissue, scalloped tongue, absence of tongue papillae and palatal rugae, microstomia, vestibule obliteration, ankyloglossia, several teeth lost due to caries	Malnutrition, chronic anemia, pseudosyndactyly, and hand retraction, recurrent urinary and wound infections, alopecia	Maxillary compression, anterior crossbite, moderate upper crowding, retroclined lower incisors, non‐coincident midlines	Hyperdivergent, Skeletal class III,	Dysphagia	History of early teeth extractions due to lack of space for upper permanent canines to erupt. Oral surgery for microstomia and vestibule obliteration.	Fixed metallic braces (MBT), Mini‐implants (22 months)	Bonding brackets in the posterior teeth was not possible due to microstomia and vestibule obliteration, using Mini‐implants as temporary anchorage.	Ulcers due to Mini‐implant	Ideal, Upper incisors rehabilitation for aesthetic improvement and occlusal stabilization,	No, 6 months later the occlusion remained stable.
Véliz, 2024 [8]	20 months to 18 years old. Intermediate JEB. Homozygous for the variant c.3228+1G>A in LAMB3	Generalized hypoplastic AI, crown resorption, gingival hyperplasia, generalized gingivitis, Restoration #21, poor oral hygiene, sensitivity due to AI	Chronic granulation areas in the scalp, alopecia, Cushing's face, nail atrophy, anemia	Maxillary compression with anterior and posterior crossbite, severe upper crowding, agenesis #45,	Skeletal class III,	—	Preventive treatment, Temporary crowns due to AI	Mini‐implants Assisted Rapid Palate Expansion (MARPE), Fixed metallic braces (MBT) (25 months: 6 months MARPE + 19 Months orthodontic)	Due to AI, it was used 5% Sodium hypochlorite to etch enamel and Assure Plus (By Reliance) to bond brackets to Acrylic crowns.	Oral ulcers due to braces, using orthodontic wax when necessary. Debonding braces due to enamel hypoplasia and acrylic crowns.	Ideal, Full mouth oral rehabilitation after orthodontic treatment.	The patient decided not to use

### 6.2 Recommendations

**1**. **General information about DO and orthodontic treatment in EB (Questions 1.1 to 1.3)**.

**1.1**. **Is orthodontic and/or DO treatment possible in patients living with EB?**

*Summary of Evidence*

In the past, several authors have stated that individuals with severe forms of EB, such as RDEB, are not suitable candidates for orthodontic treatment [6] or that orthodontic treatment is contraindicated [12]. Similarly, some members of the panel reported unsuccessful attempts to perform orthodontic treatment in patients with different types of EB.

However, successful interventions have been reported for patients with severe subtypes of EB, including severe RDEB, JEB, and KEB. The strategies implemented encompass early teeth extractions [13, 14], DO appliances [8, 15], fixed metallic braces [8, 15, 16, 17, 18], aligners [7], and mini‐implants [8, 18].

In addition to the EB‐related challenges, the American Academy of Pediatric Dentistry (AAPD) identified several variables that can affect the success of orthodontic treatment at early ages, including, but not limited to: the chronological, mental or emotional age of the patient; the ability of the patient to understand and cooperate; the intensity, frequency, and duration of an oral habit; parental support for the treatment; the compliance with the clinician's instructions; craniofacial configuration and growth; concomitant systemic disease or condition; accuracy of diagnosis; appropriateness of therapy and timing of treatment [19].

*Level of evidence: Nonanalytic studies* [level 3]

**Recommendation**:

DO and/or orthodontic treatment is possible in patients living with EB.

**BP**: Proper risk‐benefit assessment should be performed before treatment to identify variables related and not related to EB that can affect orthodontic treatment.

**1.2**. **Is orthodontic and/or DO treatment the same in complexity for all patients with EB?**

*Summary of Evidence*

Oral manifestations are very different among EB subtypes. Patients with severe forms of RDEB present strictures of the oral tissues, while those with JEB present AI, and KEB have an increased risk of periodontal disease. Reports of orthodontic treatment in patients with RDEB, JEB, and KEB reveal numerous challenges, modifications, and limitations [7, 13, 14, 15, 16, 18]. In contrast, it has been stated that patients with mild forms of EB require minor modifications and can tolerate orthodontic therapy much better [3, 6].

The article “Oral health care pathways for patients with EB: a position statement from the European Reference Network for Rare Skin Diseases” [20] classifies patients with EB into three categories according to risk for oral and dental manifestations and complications:

- Patients with high risk of oral and dental manifestations and complications (generalized and inverse types of RDEB, JEB, and KEB) present a significant level of complexity for orthodontic treatment.
- Patients with EB subtypes that are associated with a moderate risk of oral and dental manifestations and complications, such as severe EBS, dominant DEB (DDEB), and localized RDEB, present a moderate level of complexity for orthodontic treatment.
- Patients with a low risk of oral and dental manifestations and complications due to EB (EBS due to keratin, and KLHL24 mutations) present a standard complexity for orthodontic treatment.

Regardless of the complexity of the orthodontic treatment needs, the subtype of EB and its associated features will impact the complexity of the treatment.

*Level of evidence: Nonanalytic studies* [level 3]

**Recommendation**:

Orthodontic and/or DO treatment is not the same in complexity for all patients with EB.

**1.3**. **At what age should patients diagnosed with EB be referred to an orthodontist for their initial orthodontic evaluation?**

*Summary of Evidence*

It has been recommended for the general population that the initial occlusion assessment be conducted before the age of 7 years [21]. Patients with EB are expected to be referred to dental care (pediatric dentist/general dentist/special care dentist) immediately upon EB diagnosis, as early referral and close follow‐up are key to keeping patients as healthy as possible [3]. In this context, when malocclusions are noticed, patients should be referred to an orthodontist. Orthodontic and DO interventions have been reported in patients with EB as early as the age of 4 years [13, 14, 17]. The indications for orthodontic referral in special care dentistry may include, but are not limited to, malocclusion, facial compromise, and orofacial functional abnormalities, including difficulties with speech, chewing, swallowing, and breathing [9].

*Level of evidence: Nonanalytic studies* [level 3]

**Recommendation**:

Patients with EB should be referred for an orthodontic evaluation at the age of 7 or as soon as any orthodontic problems are detected.

**2**. **Orthodontic and DO diagnosis in EB (Questions 2.1 to 2.3)**.

**2.1**. **What characteristics can be observed in patients with EB seeking orthodontic treatment?**

*Summary of Evidence*

**FIGURE 1:**
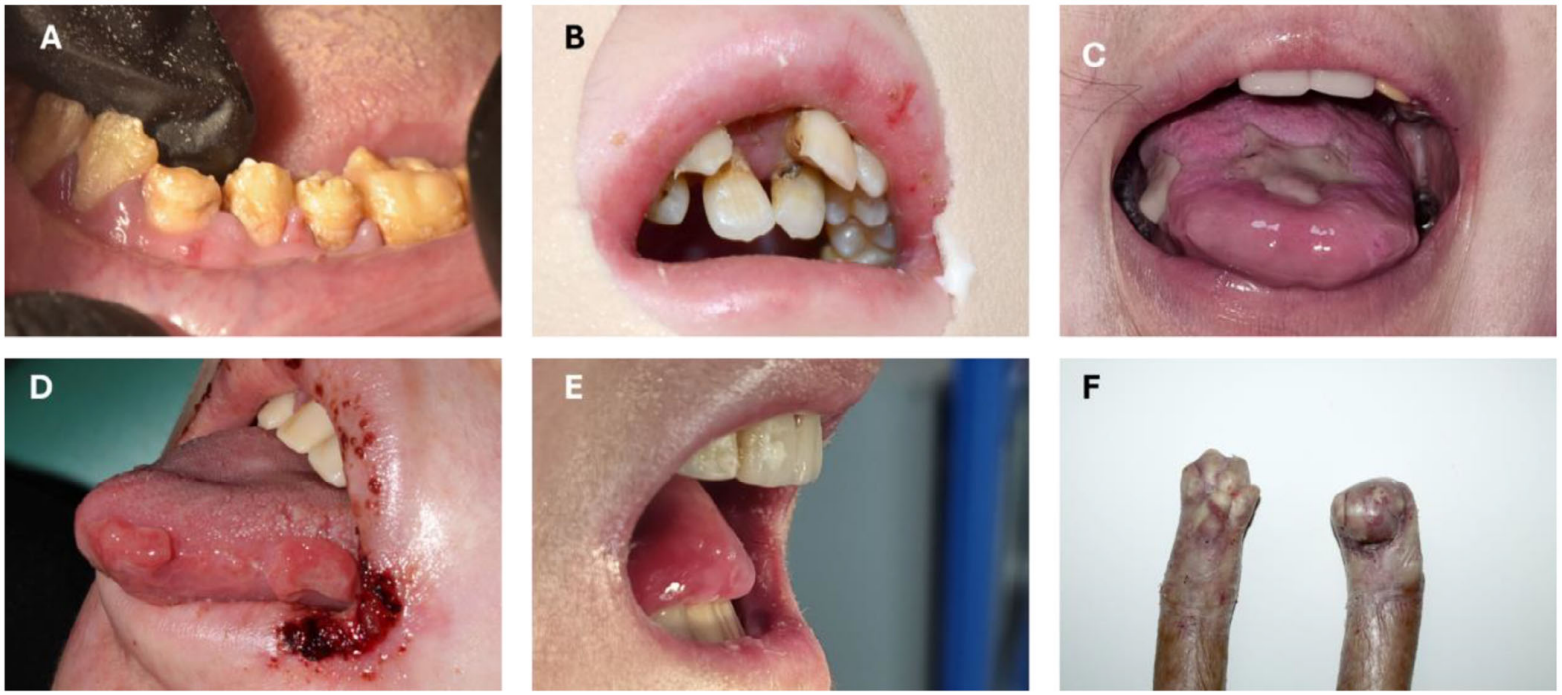
Characteristics of patients with EB subtypes with high risk of oral and dental manifestations and complications. (A) Generalized hypoplastic amelogenesis imperfecta in a patient with intermediate JEB. (B) Severe crowding and microstomia in a patient with severe RDEB. (C) Chronic intraoral granulation tissue lesion in a patient with severe JEB. (D) Perioral and intraoral granulation tissue in a patient with severe JEB. (E) Ankyloglossia and microstomia in a patient with severe RDEB. (F) Pseudosyndactyly in a patient with severe RDEB.

The phenotypic variability of EB can be attributed to the genetic variants in at least 16 distinct genes encoding structural proteins of the epidermis and the dermo‐epidermal junction. There is a wide range of clinical manifestations in different organs and systems, depending on the type and subtype [1, 2]. In addition to malocclusions, the clinical characteristics described in cases and studies on orthodontic treatment in patients with EB are reported in Table 2. These can include, but are not be limited to:

- Extraoral: Facial skin lesions such as vesicles, bullae, blisters, erosions or ulcers; facial erythema, epithelial atrophy, milia, crusts, scars, granulation tissue, angular cheilitis, and lip atrophy [7, 8, 13, 14, 15, 16, 17, 18] can be seen. Specific extraoral features are perioral granulation tissue in JEB and poikiloderma in KEB [3].
- Intraoral: Mucosal lesions such as vesicles, bullae, blisters, erosions or ulcers; granulation tissue, atrophic mucosa, scalloped tongue, oral cancer, microstomia, ankyloglossia, vestibule obliteration, caries, periodontal disease, enamel hypoplasia, absence of tongue papillae or palatal rugae [7, 8, 13, 14, 15, 16, 17, 18] (Figure 1) can be seen. In specific types, some features are:
  - RDEB: Absence of tongue papillae, vestibule obliteration, microstomia, ankyloglossia, and high caries prevalence [3].
  - KEB: Early onset periodontal diseases [5].
  - JEB: Gingival hyperplasia with profuse bleeding, AI, crown resorption during pre‐eruptive stages, eruption delay, and tooth retention [3].
- Oral functions: Pain associated with ulcers and lesions can limit orofacial functions. In RDEB, oral functions are affected by strictures, including microstomia, ankyloglossia, and vestibule obliteration, limiting mouth opening, tongue mobility, oral hygiene, deglutition, speech, swallowing, and craniofacial growth [3, 6, 22, 23, 24]
- Systemic: Body skin lesions such as vesicles, bullae, blisters, erosions or ulcers; pseudosyndactyly and musculoskeletal contractures, fine fiber neuropathy, nail atrophy, esophageal strictures, chronic granulation tissue, alopecia, recurrent infections, anemia, malnutrition, faltering growth (failure to thrive), osteopenia, kidney disease, hydrocele, urethral stenosis, constipation, vitamin D deficiency and other vitamin and mineral deficiencies, scoliosis, corneal ulcers and walking difficulties [7, 8, 13, 14, 15, 16, 17, 18].

*Level of evidence: Nonanalytic studies* [level 3]

**Recommendation**

Several clinical characteristics affect patients with EB, including extraoral and intraoral features, oral functions, and systemic conditions, especially in high‐risk EB types, such as RDEB, JEB, and KEB.

**BP**: Individual assessment should be performed to identify all extraoral and intraoral features and oral functioning characteristics that might have an impact on the diagnosis and treatment plan.

**2.2**. **What malocclusion or orthodontic diagnoses are the most common in EB patients?**

*Summary of Evidence*

In general, malocclusions have been described in patients with EB, including crowded teeth [13, 14, 15, 16, 18, 25, 26, 27], molar class II [7, 15, 16], increased overjet and overbite [7, 26], retroclined lower incisors [7], anterior, [8] and posterior crossbite [8, 25]. Skeletal classes II [7, 16] and III [18, 18] have also been identified. Some types of EB have been described to have specific dentofacial anomalies. Patients with RDEB have a reduced maxillary length, mandibular length, middle facial height, lower facial height, and distance from the lower lip to the aesthetic plane. Additionally, saddle and nasolabial angles can be greater [28]. Some authors have proposed that reduced facial growth, which is associated with malnutrition, faltering growth, and reduced alveolar arches due to soft tissue constrictions, may result in dentoalveolar disproportion and dental crowding in RDEB [3, 6]. In patients with JEB, retention of teeth (failure of eruption), crown resorption, and agenesis have been reported, which could lead to malocclusion [8, 29, 30].

*Level of evidence: Nonanalytic studies* [level 3]

**Recommendation**

The most common malocclusions described in EB are teeth crowding and posterior crossbite. Specific subtypes of EB can present distinctive features, such as reduced maxillary size in RDEB and failure of tooth eruption in JEB.

**2.3**. **Which methods are necessary to achieve a successful dentofacial orthodontic diagnosis in patients with EB?**

*Summary of Evidence*

Methods to achieve orthodontic diagnosis include [19]:

1. Clinical examination
  - General management
  - Facial examination
  - Intraoral examination
  - Functional analysis
2. Records assessment.
  - Photographs
  - Dental casts
  - Radiographs
  - Other specific complementary exams.

In patients with EB, particularly in those with a high risk of oral and dental manifestations and complications (RDEB, JEB, KEB), and some with a moderate risk (severe EBS types), these methods necessitate a modified technique or approach [3]. The evidence for these recommendations was gathered from expert opinion during the development of the present guideline.

*Level of evidence: Nonanalytic studies* [level 3] *and Expert opinion* [level 4]

**FIGURE 2:**
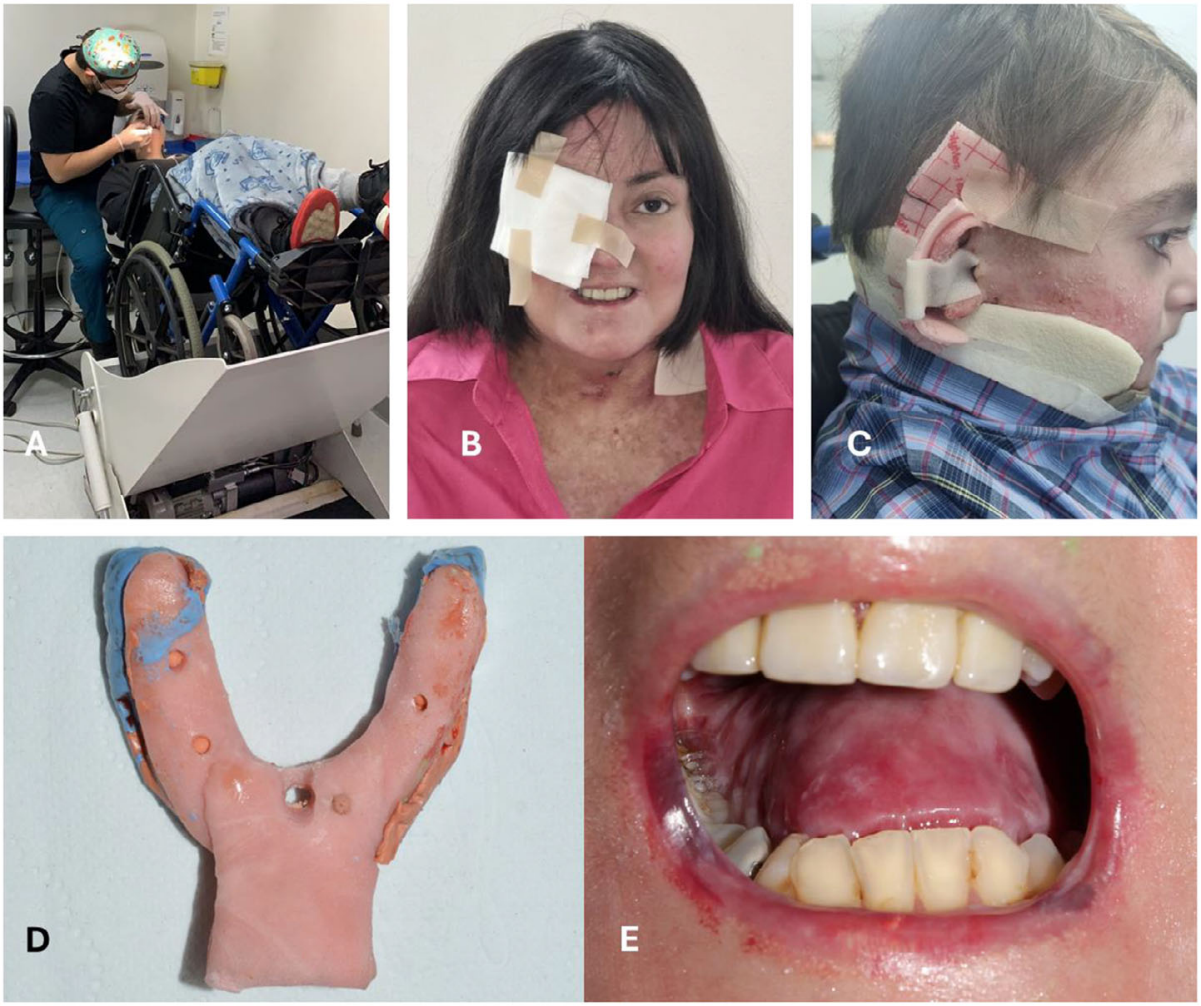
(A) A patient with RDEB, a wheelchair user, receives treatment in a wheelchair recliner. (B) An extraoral photograph of a patient with an eye ulcer covered by a dressing. (C) An extraoral photograph of a dressing covering chronic lesions on the neck and ear. (D and E) A custom‐designed acrylic lower tray was used for a patient with RDEB, but despite the efforts, a hemorrhagic lesion was produced on the right commissure.

**Recommendations Provided by the Panel of Experts**:

1. **Clinical examination**
  - General management: Patients with EB require specific skin and mucosal management, which includes the utilization of lubrication in lips, careful use of suction and air‐water syringes, employing a controlled local anesthesia technique, and oral bullae drainage. Additional padding of the dental chair may aid patients with severe RDEB in ensuring their comfort during treatment. Clinical facilities must be accessible to patients using wheelchairs (Figure 2A). If the facilities or team are not trained to treat patients with EB, educating them and creating awareness about the specific management protocols for these patients is advisable. For general management of patients with EB, refer to the CPG: Oral health care for children and adults living with EB; chapter 3: Oral health care and dental treatment for children and adults living with EB—CBG [3].
  - Facial examination: Dressings, frequent in areas such as the neck, ear, and eyes, might limit clinical examination (Figures 2B and C).
  - Intraoral examination:
    - Patients with EB of all types may present with open wounds or lesions on their lips, commissures, and oral cavity. The degree of mucosal fragility may range from extreme fragility to normal strength.
    - In patients with RDEB, an appropriate intraoral examination can be severely impaired by oral strictures (microstomia, ankyloglossia, and vestibule obliteration). Measuring teeth crowding intraorally might not be possible.
    - Patients diagnosed with JEB may exhibit areas of chronic granulation tissue, both periorally and intraorally.
    - Patients with other subtypes of EB may also face limitations according to their phenotype expression. Patients who have a history of tube feeding may experience oral‐sensory difficulties. In these patients, a multidisciplinary approach is advised with speech and language therapists.
    - Functional analysis: might be limited due to strictures, lesions, and pain.
2. Records assessment.
  - Photographs:
    - Extraoral photographs: Patients with considerable facial lesions may be reluctant to take extraoral photographs.
    - Intraoral photographs: Normal intraoral photographs with occlusal intraoral mirrors, contrasters, and lip retractors might be impossible to use without causing damage to the patient. Hence, in patients with severe contractures or fragility, only smile pictures might be possible to take. Since some patients have photophobia and a high visual sensitivity, flash should be used with caution.
  - Dental casts: In patients with severe microstomia, dental trays and intraoral scanning are difficult to fit without causing harm, mostly due to reduced mouth opening and the lack of mobility of the soft tissues (Figures 2D and E). If necessary, it is recommended to use customized or flexible standard trays [7]. Impressions with silicone can be used to obtain models from the anterior zone of the arch. In some cases, an intraoral scanner can be used only in the anterior zone, primarily because of the size of the camera and the movement required for its use.
  - Radiographs:
    - Extraoral radiographs, such as panoramic, cephalometric, or cone beams, are recommended for orthodontic treatment and do not necessitate any significant modifications. In patients with JEB, the teams must establish proper radiographic follow‐up to early identify signs of crown resorption and tooth retention. However, patient positioning should be carried out or assisted by an individual with knowledge of EB.
    - Intraoral radiographs, if needed, may be difficult to obtain due to ankyloglossia and mucosal fragility.
  - Other specific complementary exams: If dental casts are needed and it is not possible to obtain impressions or scanners, stereolithographic models can be used.

**3**. **Orthodontic and DO treatment in EB (Questions 3.1 to 3.10)**.

**3.1**. **What are the benefits of orthodontic treatment in patients with EB?**

*Summary of Evidence*

**FIGURE 3:**
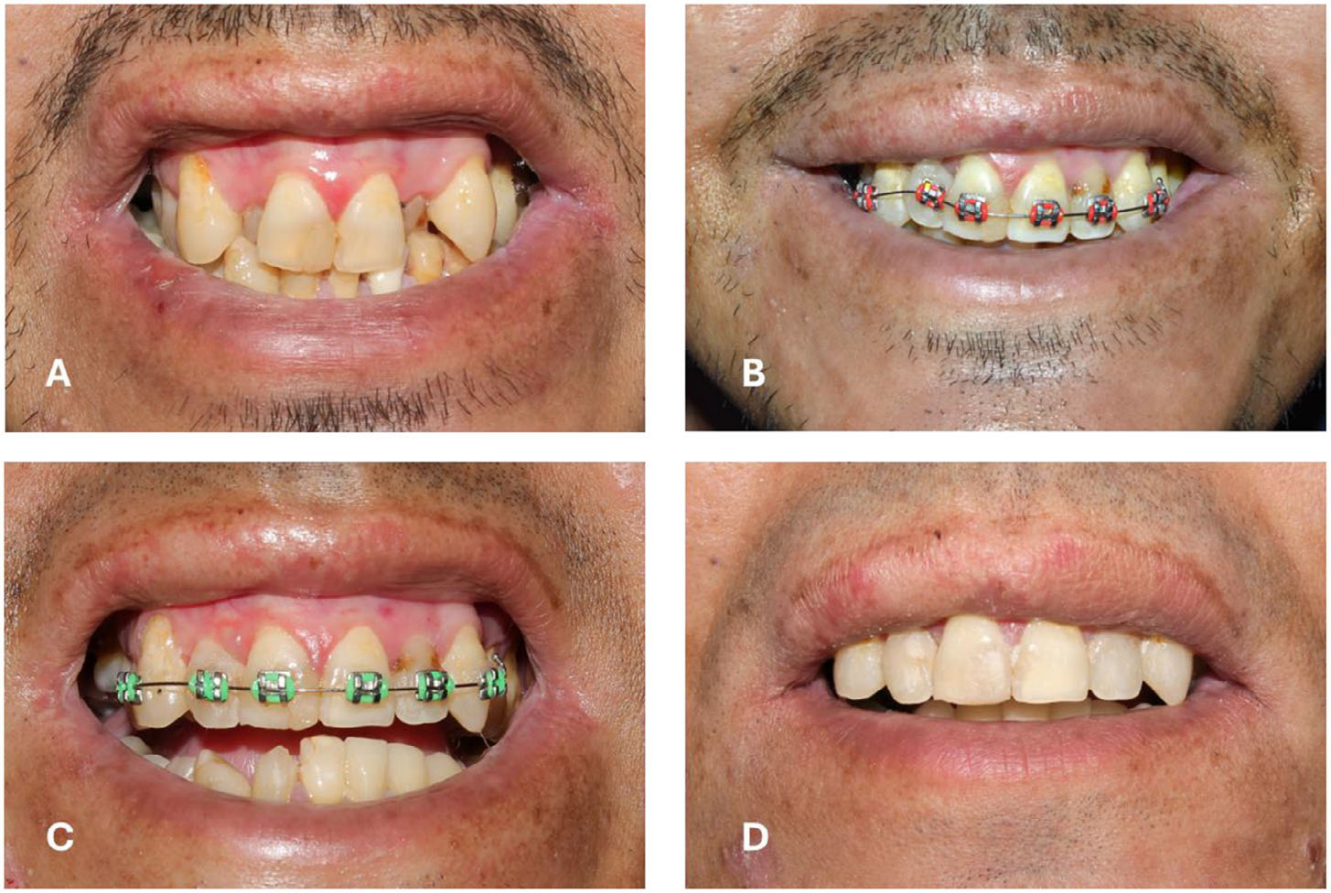
Orthodontic treatment in a patient with intermediate RDEB. (A) Severe malocclusion with an anterior crossbite in #12 and #22, both with caries, associated with difficulties in performing oral hygiene due to malposition. (B) and (C) Evolution of orthodontic treatment after 5 and 10 months, respectively. Treatment planning considered only upper arch brackets, realistic objectives (Improving upper alignment for better aesthetic and easier oral hygiene), and orthodontic strategies (Difficulty in bonding posterior tubes and brackets, meaning limited anchorage and future difficulties in space closing. Hence, the team decided not to perform first premolar extractions). (D) One year after treatment, the alignment of the anterior teeth was successful, and the lateral incisors had been restored.

The benefits reported from DO and orthodontic treatment in patients with EB include improved aesthetics and self‐perception, easier oral hygiene, and a reduction in oral ulcers, secondary to misaligned teeth [7, 8, 13, 14, 15, 16, 17, 18] (Figure 3). A patient with JEB required DO and orthodontic treatment in order to align teeth and provide sufficient space for full‐mouth oral rehabilitation with ceramic crowns [8]. A patient with RDEB reported that, after orthodontic treatment, her tongue had more space [7].

Despite the significant benefits, the panel acknowledges that orthodontic treatment in EB carries certain disadvantages, such as temporary pain and wounds, and an increased risk of caries during treatment, which can result in significant tooth structure loss if not properly managed. A risk‐benefit analysis should be performed for each patient [3].

*Level of evidence: Nonanalytic studies* [level 3]

**Recommendation**:

Orthodontic treatment has the potential to benefit patients with EB. The reported benefits include enhanced aesthetics, easier oral hygiene, and fewer traumatic ulcers in well‐aligned teeth. Before orthodontic treatment, a proper risk‐benefit assessment is mandatory. During orthodontic treatment, patients can present transitory pain, wounds, and an increased risk of caries due to difficulties performing oral hygiene with fixed appliances.

**3.2**. **What are the multidisciplinary areas that patients with EB may require prior to, during, or subsequent to receiving orthodontic treatment?**

*Summary of Evidence*

Before beginning orthodontic treatment, a comprehensive treatment plan should be discussed with all members of the multidisciplinary team. The team may comprise the patient's lead dentist (either a general dentist, pediatric dentist, or special care dentist) [3], the orthodontist, and other professionals, depending on the patient's requirements.

**FIGURE 4:**
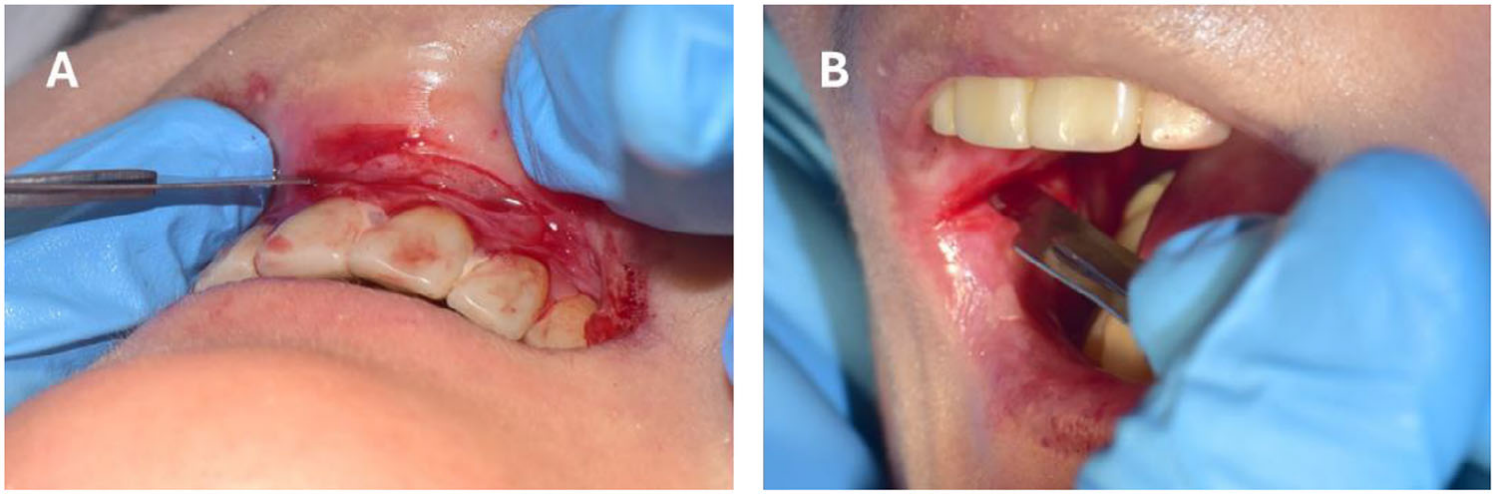
Stricture‐release surgery before taking impressions for the aligners. (A) Vestibuloplasty (B) Mucosal stricture‐release surgery to increase mouth opening.

For patients with severe strictures, oral surgeons should be considered to determine the management of microstomia, vestibule obliteration, ankyloglossia, and the need for temporary anchorage with mini‐implants [7, 18] (Figure 4). Speech therapists must assess orofacial functions in all patients with EB, particularly those who have functional limitations due to oral strictures and those who have received parenteral nutrition [22, 23]. In patients with oral‐sensory difficulties, a speech and language therapist should be invited [31, 32]. In patients with syndromic AI associated with JEB, an oral rehabilitation specialist should be involved in the treatment planning process from the initial stages of treatment [8]. It may be necessary to schedule frequent periodontal appointments due to poor oral hygiene [8, 18], particularly in patients with KEB due to early onset periodontal diseases [17].

*Level of evidence: Nonanalytic studies* [level 3]

**Recommendation**

Orthodontic treatment requires a multidisciplinary approach, including special care dentistry, pediatric dentistry, oral rehabilitation, periodontology, and speech therapy. Additional specialties that may need to be part of the team can be, but are not limited to: maxillofacial surgery, dermatology, pediatrics, nutrition, and psychology.

**3.3**. **Which considerations should be taken into account when planning orthodontic treatment for patients with EB?**

*Summary of Evidence*

No specific information on orthodontic planning for patients with EB was found in the literature. Most cases reported the outcomes of every treatment, but very few considerations regarding treatment planning. An important part of the evidence for these recommendations was gathered from expert opinion during the development of the present guideline.

*Level of evidence: Nonanalytic studies* [level 3] *and Expert opinion* [level 4]

**Recommendations Provided by the Panel of Experts**

**FIGURE 5:**
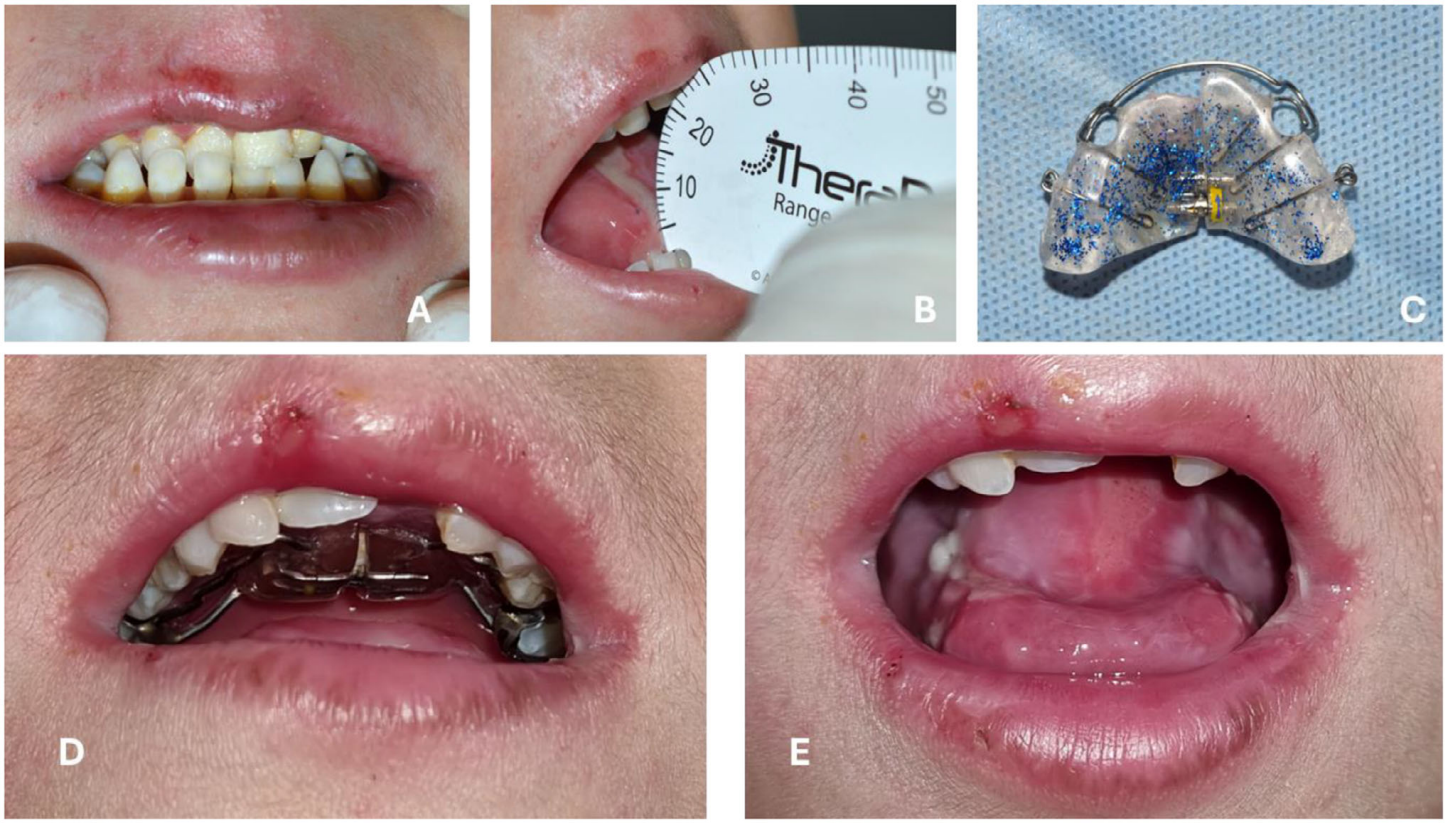
(A) Male patient with intermediate RDEB at the age of 5 years presented anterior and posterior crossbite. (B) Maximal mouth opening 20 mm. (C) Early intervention was planned. Dental casts were obtained by using a custom‐made acrylic tray. A removable expansion appliance was conceived. The patient did not like using the appliance and never wore it. The treatment was suspended because of a lack of compliance. (D) The family made a request for a second treatment attempt at the age of 8. This time, a fixed appliance was planned, designed, and bonded. (E) The patient refused to eat with the appliance in the mouth. A week after bonding, the appliance had to be removed. Treatment was stopped for the second time.

- Orthodontic planning for patients with EB must be realistic and patient‐centered.
- The treatment objectives must be realistic in light of the severity of the malocclusion and adapted to the orofacial and psychosocial implications of EB. Common treatment objectives include achieving tooth alignment, improving aesthetic complaints, enhancing transverse maxillary dimension, and providing occlusal stability [8, 18].
- Treatment should be planned in stages, continuing to the next stage once the objective has been achieved.
- The duration of treatment in RDEB patients is significantly longer, both during each session in the dental chair and throughout the entire treatment process, compared to other EB and non‐EB patients.
- Therapeutic alliance and behavioral support are important and cannot be overstated, as orthodontic treatment can extend for several years. Behavioral support techniques can include pharmacological techniques (sedation) for some specific clinical procedures, such as long sessions for bracket bonding or mini‐implant positioning. Still, they are not recommended for routine treatment sessions, such as regular orthodontic visits [3].
- In some cases, the patient or the clinical team may decide to interrupt or stop treatment. Patients may wish to discontinue the treatment due to discomfort or ulcers associated with the appliances. Clinicians may indicate to pause or stop the treatment because of poor oral hygiene with an increased number of caries, or when the patient presents additional systemic complications that make it difficult to attend the routine monthly session. In these cases, all orthodontic appliances should be removed and, if possible, the occlusion should be stabilized with aligners or thermoplastic devices (Figure 5).

3.4
**Which adjustments can be implemented to adapt orthodontic techniques for patients with EB?**

*Summary of Evidence*

Reports have shown that DO and orthodontic treatment can be provided for patients with EB with a variety of appliances. Depending on the patient's oral features, different adjustments can be implemented to adapt orthodontic techniques for patients with EB (Table 2). The adjustments described in the literature have been complemented with the expert opinion during the development of the present guideline.

*Level of evidence: Nonanalytic studies* [level 3] *and Expert opinion* [level 4]

**Recommendations**

Orthodontic treatment may benefit from specific adjustments depending on the phenotypic expression of each subtype of EB, especially in RDEB, KEB, and JEB.

**FIGURE 6:**
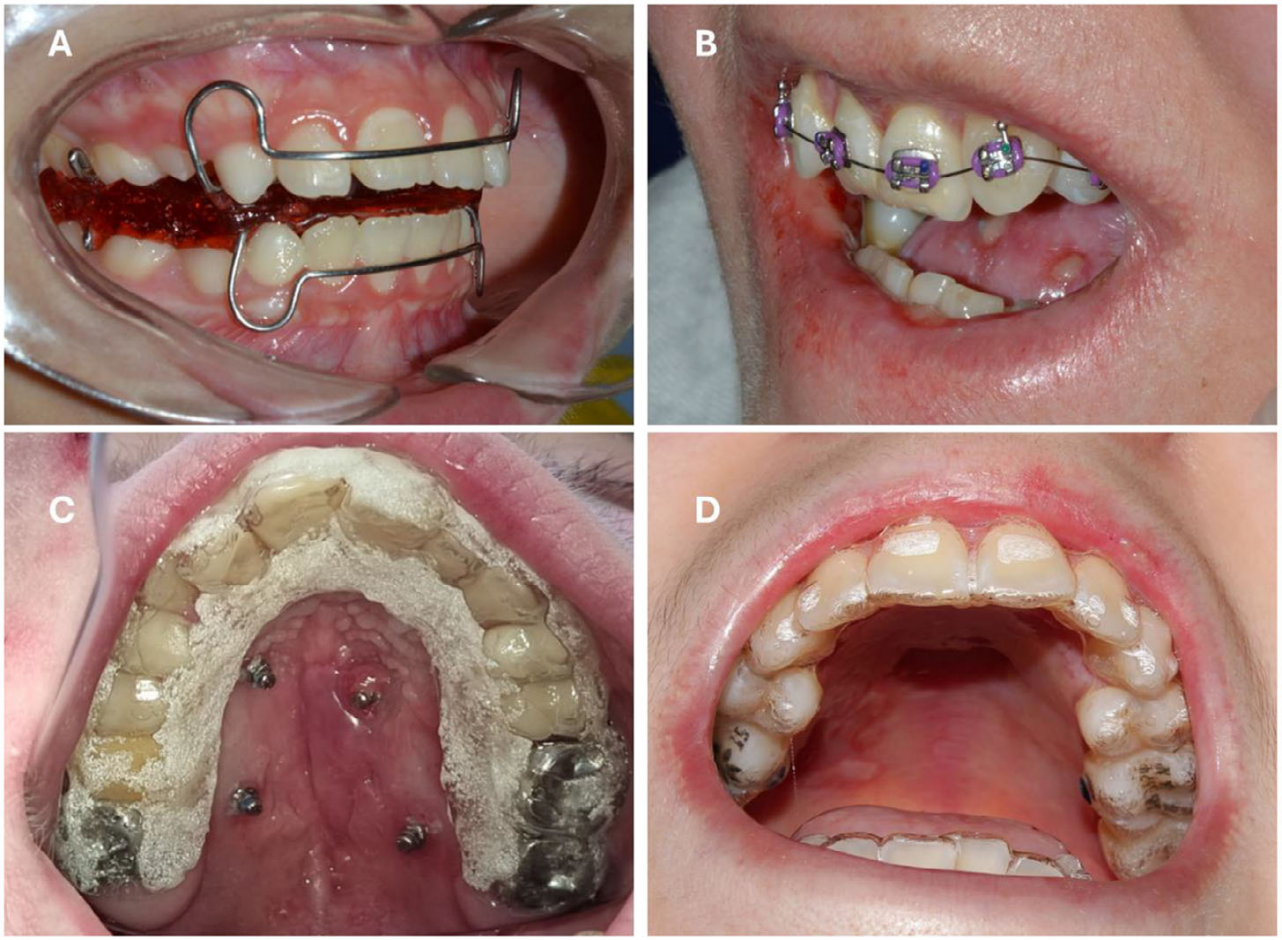
(A) A removable dentofacial orthopedic appliance in a patient with localized DDEB. (B) Fixed metallic brackets in a patient with severe RDEB. (C) A patient with intermediate JEB with four mini‐implants placed on the palate for bonding a MARPE appliance (Mini‐implant assisted rapid palate expansion). On the occlusal surfaces a temporary retention device is used. (D) Aligners in a patient with intermediate RDEB.

- Early/selective tooth extraction: As patients with severe RDEB present reduced alveolar arches leading to severe crowding, it has been suggested to perform early/selective tooth extractions to guide the eruption process into a better‐aligned arch. Reported experiences have successfully improved tooth crowding and oral hygiene after the extraction of the first premolars. Uncommon extraction patterns have also been reported, such as a patient with severe microstomia (mouth opening of 6 mm) in whom both upper permanent canines were extracted due to severe lack of space, limited access to continuous dental treatment, and already well‐aligned lateral incisors and premolars [13, 14].
- Removable DO appliances: Taking an impression can be challenging in patients with severe microstomia. In question 2.3, section 2b of the present document, different methods for taking impressions, such as custom‐made or flexible trays, intraoral scanners, and stereolithography, are described [7]. Modifications of the appliance include covering the retentive structures with acrylic, which can reduce the risk of wounds due to metallic structures such as clasps and springs [33]. In the case of removable appliances, these must be tooth‐worn, as the pressure and pain associated with mucosal support might reduce compliance with the therapy [3] (Figure 6A.
- Fixed DO appliances: Fixed palatal expanders, such as Hyrax, can be used in EB. Metallic bands, bonded acrylic expanders, or mini–implants can be used for anchorage. One of the limitations of the technique is the difficulty in obtaining impressions and adapting the bands. There is not enough evidence to support one anchorage technique over the other. To select the proper technique it is important to consider the potential discomfort associated with the expansion protocol and the possibility of ulcers arising from the interaction between the Hyrax and the mucosa. Ideally, mucosal support should be avoided [3, 8, 13]. Bite ramps are also used to manage occlusion during mixed dentition, especially in the anterior teeth.
- Fixed braces: Metallic fixed braces, whether regular or self‐ligating, are a treatment alternative in patients with different subtypes of EB, including RDEB. Characteristics such as microstomia, vestibule obliteration, ankyloglossia, and mucosa fragility may make the bonding technique difficult, especially in the posterior area. As wire changes can cause ulcers and pain, some authors have suggested limiting the orthodontic treatment to the anterior teeth [18]. If brackets are planned on the anterior teeth only, orthodontic anchorage on the posterior area will be restricted, making it challenging to perform retrusive movements. In other words, tooth extraction to retrude anterior teeth should only be performed if posterior anchorage has been successfully secured [18]. Other examples of adjustments to facilitate treatment include performing a vestibuloplasty prior to treatment in patients with severe contractures [7] or removing the hook from the brackets/bands [3]. Patients with JEB and AI may also encounter bonding difficulties owing to the altered enamel structure or the restored teeth (metallic, polycarbonate, acrylic, and ceramic crowns, composite, and glass Ionomer). In cases of hypoplastic AI, the bonding technique might require adjustments. An example reported in the literature is the use of 5% sodium hypochlorite as an etching agent and All‐Surface bonding agents (Assure‐Plus by Reliance) [8] (Figures 6B).
- Mini‐implants: Successful use of mini‐implants has been reported in RDEB and JEB patients. They were used to reinforce the anchorage with fixed braces and as anchorage to perform maxillary rapid palate expansion (MARPE). The protocols used were similar to the general population; however, the patient with RDEB required surgery to increase mouth opening and vestibule depth before placing the mini‐implants [8, 18] (Figures 6C).
- Aligners: Successful use of aligners has been reported in RDEB. Nevertheless, adjustments were necessary to obtain the impression, as microstomia limited intraoral scanning. An impression made using adhesion elastomeric silicone on a flexible tray allowed to obtain a cast, additionally, digital improvement of silicone defects was conducted prior to printing the aligners [7]. When planning aligner therapy, it is important to design the treatment trying to consider attachments in the anterior area mainly, as bonding is difficult and challenging in the posterior region of those patients with oral contractures. The risk of caries should be considered when stripping in RDEB (Figures 6D).

**FIGURE 7:**
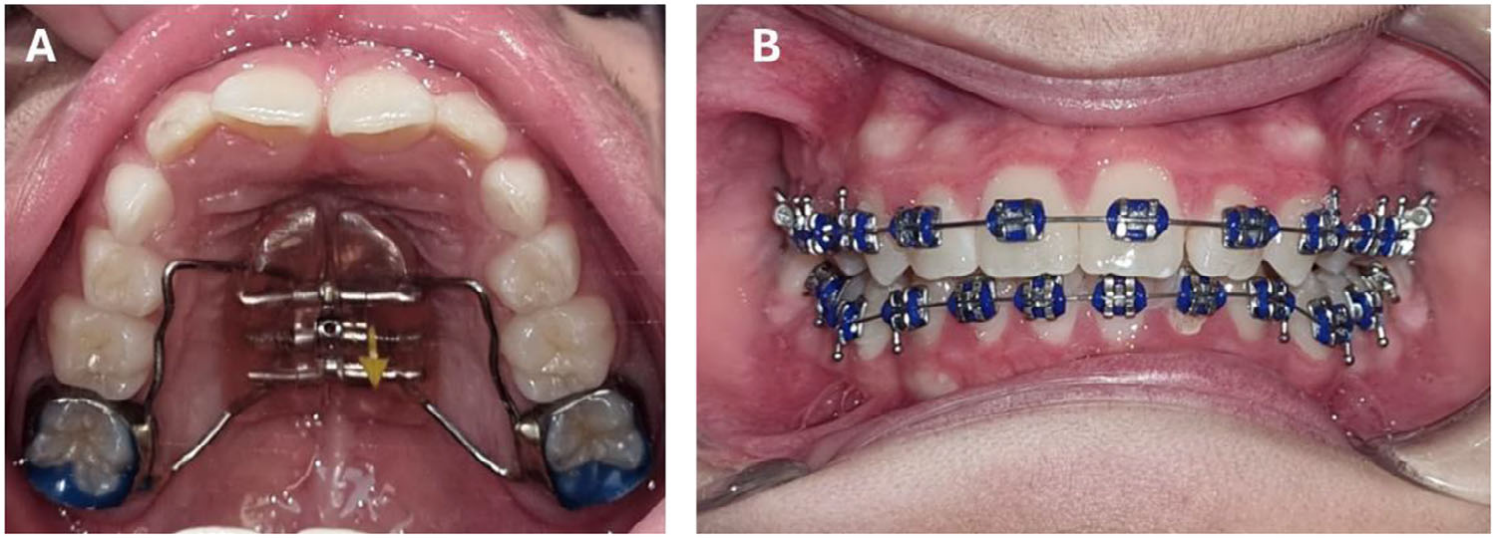
Patient with moderate risk of oral manifestations (DDEB) during (A) Dentofacial orthopedic and (B) orthodontic treatment. The treatment followed the protocols used for non‐EB patients. The patient occasionally utilized orthodontic wax.

**3.5**. **Is orthodontic retention necessary for individuals with EB?**

*Summary of Evidence*

The use of orthodontic retention is highly recommended, especially in patients with RDEB, due to relapse associated with an imbalance of orofacial forces caused by the constrictions. Fixed retention may be challenging due to the elevated risk of caries, difficult bonding technique, and tongue lesions if the surface is poorly polished. Conversely, removable devices may be difficult to obtain due to microstomia but have been previously used in EB [15, 16]. If retention is not used, a relapse of the malalignment can be observed.

*Level of evidence: Nonanalytic studies* [level 3] *and Expert opinion* [level 4]

**Recommendation**

Orthodontic retention is highly recommended, especially for patients with a functional imbalance or a high risk of malocclusion relapse.

**BP**: The retention types should be selected according to the clinical features, feasibility, and risk of caries.

**3.6**. **What are the complications associated with orthodontic treatment in individuals diagnosed with EB?**

*Summary of Evidence*

The complications most reported by patients with EB are traumatic ulcers, pain, caries associated with poor oral hygiene and brackets debonding. It is expected that patients with EB present more ulcers than regular orthodontic patients due to mucosal fragility, resulting in pain and discomfort (Figures 5E). The management of oral ulcers will depend on the patient's tolerance, ranging from using orthodontic wax, mouthwashes aimed at enhancing healing of oral ulcers, to suspension of the treatment. Despite the severe general compromise caused by EB, certain patients exhibit exceptional tolerance toward the ulcers caused by the brackets. On the other hand, some patients with a low history of oral lesions may experience difficulties coping with the pain associated with both oral ulcers and the pain caused by the treatment.

Patients with RDEB and a high risk of caries should be closely monitored, as this may escalate even further during orthodontic treatment. Debonding can be frequent in EB types with enamel hypoplasia [3, 8, 17].

*Level of evidence: Nonanalytic studies* [level 3] *and Expert opinion* [level 4]

**Recommendation**

Complications associated with orthodontic treatment in individuals diagnosed with EB may include, but are not limited to, oral ulcers, pain, poor oral hygiene with an increased number of caries, and recurrent debonding of braces.

**3.7**. **What considerations are necessary for maintaining oral hygiene in patients with EB who are undergoing orthodontic treatment?**

*Summary of Evidence*

Oral hygiene can be difficult for patients with EB, even without orthodontic appliances, due to pseudosyndactyly, microstomia, ankyloglossia, vestibule obliteration, mucosal fragility, perioral granulation tissue, and pain [3]. Consequently, oral hygiene during DO or orthodontic treatment is even more complex, requiring adjustments, complementary tools, carer education, and monitoring of oral hygiene, and frequent professional hygiene. Dental floss can be challenging to use, especially for patients with pseudosyndactyly and braces, who require assistance from carers to use it. Patients with sensory issues may have difficulties with tooth brushing and toothpaste flavors [3].

The panel has used the following complementary products: special toothbrushes with curved, soft bristles (Collis Curve, Dr. Barman's Superbrush,), water flossers, interproximal brushes, orthodontic wax or similar products to avoid traumatic ulcers (Gishy Goo), high fluoride or bioactive paste (Duraphat, GC Tooth Mousse) or mouthwash (Xylitol, Dentoxol), plaque indicators and probiotics (Bactoblis).

*Level of evidence: Nonanalytic studies* [level 3] *and Expert opinion* [level 4]

**Recommendation**

- Oral hygiene of patients with EB undergoing orthodontic treatment is more complex and requires precise guidance for both the patients and/or their carers.
- The brushing technique should be complemented with specific hygiene elements and products. In children and adults with limited manual dexterity, it is advisable that the carers assist the brushing technique.
- Frequent professional hygiene and preventive measures, such as fluoride varnish, must be carried out by the patient's general dentist.

**3.8**. **What are the barriers patients with EB face when it comes to orthodontic treatment?**

*Summary of Evidence*

Patients with EB can face numerous barriers when it comes to accessing orthodontic treatment. Some are related to the disease itself (pain, wounds, anxiety, systemic health status) and others to the patient's social environment (funding, long travel distances to the reference centers). Barriers related to the orthodontist include the low availability of professionals in each region, insufficient training in rare diseases, and fear of treating/harming patients with EB.

One alternative to address these barriers is implementing nationally funded reference centers where knowledgeable professionals treat patients with rare diseases. These centers should be multidisciplinary, including orthodontists.

Barriers related to the availability of orthodontists with knowledge on EB should be addressed by adding lectures on special care and rare diseases to the orthodontist graduate programs, as well as promoting conferences on these topics at local and regional orthodontic conferences. Open‐access clinical guidelines on specific rare diseases, such as the present document, are also a tool to reduce barriers to care and knowledge.

*Level of evidence: Expert opinion* [level 4]

**Recommendation**

Patients with EB who need orthodontic treatment can face barriers due to their EB, such as pain, wounds, anxiety, and other associated systemic complications; and barriers in their social environment, such as funding and availability of specialized services locally. From a professional perspective, the lack of training in rare diseases among orthodontists can be the main barrier. To overcome these barriers, nationally funded reference centers could be an aid in improving access to orthodontics for patients with EB. Local barriers must be identified and addressed.

**3.9**. **What are the adjustments for orthodontic treatment needed by patients with EB subtypes with low and moderate risk of oral and dental manifestations and complications?**

*Summary of Evidence*

The classification of EB is based on the level of skin cleavage observed on a biopsy, not on the severity of the disease. However, for easier understanding of the clinical adjustments needed by every patient, Kramer et al. proposed a division into three degrees of severity according to the risk of oral and dental manifestations and complications [20]. Patients with moderate risk include those with a diagnosis of *severe EBS*, *DDEB*, and *localized RDEB*, while low risk has been established for patients with *localized and intermediate EBS* due to keratin and *KLHL24* mutations.

No data on the risk of malocclusion in patients from this group was found, and no cases were reported in the literature. As the main orofacial feature observed in these groups of patients is localized skin and mucosal fragility, without severe contractures or enamel abnormalities. The orthodontic diagnosis and treatment of these patients will need adjustments according to their individual phenotypic expression (Figure 7). In general, protocols for non‐EB patients can be applied, considering oral blisters and ulcers as needed.

*Level of evidence: Expert opinion* [level 4]

**Recommendation**

Patients with EB with low and moderate risk of oral manifestations will need adjustments according to their individual phenotypic expression, considering oral blisters and ulcers. In general, protocols for non‐EB patients can be applied.

**4**. **Other groups’ perspectives on this guide (Questions 4.1)**.

**4.1**. **What are the perspectives of patients and other members of the medical team regarding this CPG?**

Patients and other members of the medical team were invited to share their opinions and experiences regarding orthodontic treatment for people living with EB. Quotes from the participants can be found in Table S4.

The participants agreed that it is possible to perform orthodontic treatment in people living with EB, and this can improve oral function and quality of life. They also acknowledge the necessity of a multidisciplinary approach that includes orthodontists in the oral health and EB team. Several barriers were mentioned, including pain and complications caused by the treatment, limited access, and lack of professionals trained in EB, lack of scientific evidence, and economic and institutional support in this area. Consequently, there are still numerous challenges associated with this area of oral health.

## 7 Discussion

People living with rare diseases present a high prevalence of malocclusions, dental‐skeletal abnormalities, and oromotor dysfunction. Orthodontic treatment can be successfully performed in many cases; however, their access to orthodontic treatment may be limited by several barriers related to the patient, the carers, the guardians, or the practitioners [34]. For patients living with EB, oral healthcare is a major component of multidisciplinary management [3]. Malocclusions are frequent and can be treated; however, access to care remains challenging (Tables 1 and 2).

Recently, referral pathways for oral care in EB were published, highlighting the role of early orthodontics in patients with RDEB and JEB due to their high risk of oral disease [20]. This CPG is the result of a collaborative effort of different actors involved in the care of people living with EB, including patients, dental specialists (orthodontists, pediatric dentists, and special care dentists), and other members of the medical team (dermatologists, speech therapists, and psychologists). Patients' and their representatives’ perspectives were also important to include, as their views might differ from the professionals’ opinion [35]. This document aims to be an enabler/facilitator of orthodontic treatment for people with EB, providing guidance and decreasing barriers to access for this specific group.

Professionals providing orthodontic treatment should be part of the dental team treating patients with EB, understanding the different aspects of this condition and how their treatment should be adapted to the variety of clinical features experienced. The panel agreed that not all patients with EB will benefit from orthodontic treatment, as it can cause complications such as wounds or pain. However, patients with a favorable risk/benefit assessment can improve their oral health, their orofacial functions and their oral health related QoL, as orthodontic treatment can impact their self‐esteem and confidence. The diversity in the background and countries of the participants shows that, despite the different healthcare systems and providers, orthodontic treatment can be performed in patients with EB.

A limitation of this CPG is that the evidence on orthodontic treatment is scarce. Most recommendations resulting from this CPG were made considering experts’ opinions and clinical case reports, with only a few studies available [22, 23, 24, 25, 27, 28]. The low number of external reviewers can also be considered a limitation. This is explained because EB is a rare disease, and only a limited number of experienced clinicians were identified, all of whom were invited to be part of the panel; therefore, no more possible reviewers were identified.

## 8 Conclusions

Orthodontic treatment guidelines for patients living with EB are presented, including general aspects of EB, orthodontic diagnosis, and orthodontic treatment. DO and orthodontic treatment can be successfully performed in patients living with EB. A proper assessment must be performed, including a risk/benefit ratio, as not all patients will benefit from it. Orthodontic treatments will require adjustments depending on the phenotypic expression, EB type, and subtype.

## Author Contributions

Conceptualization: Sebastián Véliz and Susanne Kramer. Methodology: Sebastián Véliz, Susanne Kramer, and María Teresa Abeleira. Validation: Sebastián Véliz and Susanne Kramer. Formal analysis: Sebastián Véliz and Susanne Kramer. Investigation: Sebastián Véliz, María Teresa Abeleira, María Concepción Serran, Carole Charavet, Kirsten FitzGerald, Francisca Hormazábal, Hinrich Huber, María Korolenkova, Suhaym Mubeen, Sanchit Paul, Gabriela Scagnet, Lorena Sepúlveda, Elena Syomkina, Svetlana Tekucheva, An Verdonck, Anne W Lucky, Cristina Has, Gudrun Salamon, and Susanne Kramer. Data curation: Sebastián Véliz and Susanne Kramer. Writing‐Original Draft: Sebastián Véliz and Susanne Kramer. Writing—Review and editing: Sebastián Véliz, María Teresa Abeleira, María Concepción Serran, Carole Charavet, Kirsten FitzGerald, Francisca Hormazábal, Hinrich Huber, María Korolenkova, Suhaym Mubeen, Sanchit Paul, Gabriela Scagnet, Lorena Sepúlveda, Elena Syomkina, Svetlana Tekucheva, An Verdonck, Anne W Lucky, Cristina Has, Gudrun Salamon, and Susanne Kramer. Visualization: Sebastián Véliz and Susanne Kramer. Supervision: Susanne Kramer.

## Ethical Statement

Ethical approval was obtained from the Ethics Committee of the North Metropolitan Health Service, Chile (002/2023).

## Conflicts of Interest

The authors do not declare any conflict of interest, and none have any connection to manufacturers.

## Consent for Publication

The participant patients signed an informed consent to include their photos and information in this article for publication.

## Supporting information




**Supplementary Table 1**: List of participants 
**Supplementary Table 2**: List of Recommendations and agreement Round 1 
**Supplementary Table 3**: List of Recommendations and agreement Round 2 
**Supplementary Table 4**: Patients and non‐dentist healthcare professionals perspectives

## Data Availability

The corresponding author will share the data underlying this article upon a reasonable request.

## References

[1] C. Has , J. W. Bauer , C. Bodemer , et al., “Consensus Reclassification of Inherited Epidermolysis Bullosa and Other Disorders With Skin Fragility,” British Journal of Dermatology 183, no. 4 (2020): 614–627. 32017015 10.1111/bjd.18921

[2] A. Bardhan , L. Bruckner‐Tuderman , I. L. C. Chapple , et al., “Epidermolysis Bullosa,” Nature Reviews Disease Primers 6, no. 1 (2020): 78. 10.1038/s41572-020-0210-0 32973163

[3] S. Krämer , J. Lucas , F. Gamboa , et al., “Clinical Practice Guidelines: Oral Health Care for Children and Adults Living With Epidermolysis Bullosa,” Special Care in Dentistry 40, no. S1 (2020): 3–81. 10.1111/scd.12511 PMC7756753 33202040

[4] C. Besa‐Witto , A. Ortega‐Pinto , S. Véliz , et al., “Prevalence of Crown Resorption in Amelogenesis Imperfecta due to Junctional Epidermolysis Bullosa,” Oral Diseases 31, no. 6 (2025): 1900–1908. 39777984 10.1111/odi.15250 PMC12291425

[5] S. Krämer , A. L. Hillebrecht , Y. Wang , et al., “Orofacial Anomalies in Kindler Epidermolysis Bullosa,” JAMA Dermatology 160, no. 5 (2024): 544–549. 38506824 10.1001/jamadermatol.2024.0065 PMC10955352

[6] J. T. Wright , J. D. Fine , and L. Johnson , “Hereditary Epidermolysis Bullosa: Oral Manifestations and Dental Management,” Pediatric Dentistry 15, no. 4 (1993): 242–248. 8247897

[7] S. Véliz Méndez , M. Baeza , and S. Krämer Strenger , “Impression Technique Modification and Oral Contracture Release Surgery for Orthodontic Treatment in a Patient With Severe Microstomia due to Recessive Dystrophic Epidermolysis Bullosa,” Special Care in Dentistry 43, no. 5 (2023): 689–695, 10.1111/scd.12808. 36504454

[8] S. Véliz , A. Olivares , and S. Krämer , “Mini‐Implant Assisted Palate Expansion and Digital Design in Junctional Epidermolysis Bullosa and Amelogenesis Imperfecta: Case Report,” Special Care in Dentistry 44, no. 6 (2024): 1572–1580, 10.1111/scd.13044. 39034598

[9] American Academy of Pediatric Dentistry , “Management of Dental Patients With Special Health Care needs,” in The Reference Manual of Pediatric Dentistry (American Academy of Pediatric Dentistry, 2024), 343–350.

[10] Scottish Intercollegiate Guidelines Network (SIGN) , A Guideline Developer's Handbook (SIGN, 2025), (SIGN publication no. 50), http://www.sign.ac.uk.

[11] M. C. Brouwers , K. Kerkvliet , and K. Spithoff , AGREE Next Steps Consortium , “The AGREE Reporting Checklist: A Tool to Improve Reporting of Clinical Practice Guidelines,” Bmj 352 (2016): i1152, 10.1136/bmj.i1152. 26957104 PMC5118873

[12] A. Polizzi , S. Santonocito , R. Patini , et al., “Oral Alterations in Heritable Epidermolysis Bullosa: A Clinical Study and Literature Review,” BioMed research international 2022 (2022): 6493156, 10.1155/2022/6493156. 35686231 PMC9173894

[13] E. Portillo Nava , T. Ángeles E de la , and A. Durán Gutiérrez , “Manejo estomatológico de la maloclusión Dental en los Pacientes con epidermólisis bullosa Distrófica Mediante la Guía Interceptiva de la Oclusión (GIO): Comparación de Dos Casos,” Revista Mexicana de Ortodoncia 2, no. 2 (2014): 114–121.

[14] S. Véliz , H. Huber , M. J. Yubero , I. Fuentes , F. Alsayer , and S. M. Krämer , “Early Teeth Extraction in Patients With Generalized Recessive Dystrophic Epidermolysis Bullosa: A Case Series,” Special Care in Dentistry 40, no. 6 (2020): 561–565. 32880999 10.1111/scd.12515

[15] W. Pacheco and R. Marques de Sousa Araugio , “Orthodontic Treatment of a Patient With Recessive Dystrophic Epidermolysis Bullosa: A Case Report,” Special Care in Dentistry 28, no. 4 (2008): 136–139. 18647373 10.1111/j.1754-4505.2008.00028.x

[16] F. Goldschmied , “Orthodontic Management of a Patient With Epidermolysis Bullosa,” Australian Orthodontic Journal 15, no. 5 (1999): 302–307. 10806937

[17] I. Blanchet , C. Tardieu , and E. Casazza , “Oral Care in Kindler Syndrome: 7‐Year Follow‐Up of 2 Brothers,” Journal of Clinical Pediatric Dentistry 45, no. 1 (2021): 41–47. 33690828 10.17796/1053-4625-45.1.8

[18] S. Véliz Méndez , M. Baeza Paredes , A. Olivares , M. J. Vicuña , and S. M. Krämer Strenger , “Comprehensive Orthodontic Treatment Using Miniscrews and Digital Rehabilitation in a Patient With Severe Recessive Dystrophic Epidermolysis Bullosa,” Special Care in Dentistry 44, no. 3 (2024): 779–786, 10.1111/scd.12947. 38054659

[19] American Academy of Pediatric Dentistry , “Management of the Developing Dentition and Occlusion in Pediatric dentistry,” in The Reference Manual of Pediatric Dentistry (American Academy of Pediatric Dentistry, 2024), 475–493.

[20] S. Krämer , A. L. Hillebrecht , K. Bekes , et al., “Oral Health Care Pathways for Patients With Epidermolysis Bullosa: A Position Statement From the European Reference Network for Rare Skin Diseases,” Journal of the European Academy of Dermatology and Venereology 39, no. 6 (2025): 1080–1090. 39673192 10.1111/jdv.20498 PMC12105416

[21] I. Pietila , T. Pietila , P. Pirttiniemi , J. Varrela , and P. Alanen , “Orthodontists″ views on Indications for and Timing of Orthodontic Treatment in Finnish Public Oral Health Care,” European Journal of Orthodontics 30, no. 1 (2007): 46–51. 17962314 10.1093/ejo/cjm085

[22] C. Stellingsma , P. U. Dijkstra , J. Dijkstra , J. C. Duipmans , M. F. Jonkman , and R. Dekker , “Restrictions in Oral Functions Caused by Oral Manifestations of Epidermolysis Bullosa,” European Journal of Dermatology 21, no. 3 (2011): 405–409. 21609900 10.1684/ejd.2011.1356

[23] A. A. Poberezhnaya and M. V. Korolenkova , “Age‐Related Dynamics of Mouth Opening and Tongue Mobility in Children With Various Forms of Epidermolysis Bullosa,” Stomatologiya 100, no. 1 (2021): 55. 10.17116/stomat202110001155 33528957

[24] M. V. Korolenkova and A. A. Poberezhnaya , “Morphological and Functional Assessment of the Oral Mucosa in Children With Dystrophic Epidermolysis Bullosa,” Stomatologiya 101, no. 2 (2022): 63. 10.17116/stomat202210102163 35362705

[25] M. De Benedittis , M. Petruzzi , G. Favia , and R. Serpico , “Oro‐Dental Manifestations in Hallopeau‐Siemens‐Type Recessive Dystrophic Epidermolysis Bullosa,” Clinical and Experimental Dermatology 29, no. 2 (2004): 128–132. 14987265 10.1111/j.1365-2230.2004.01485.x

[26] C. Serrano Martínez , F. J. Silvestre Donat , J. V. Bagán Sebastián , M. Peñarrocha Diago , and J. J. Alió Sanz , “Epidermólisis ampollosa Hereditaria a Propósito del Manejo Odontológico de Tres Casos Clínicos,” Medicina Oral 6, no. 1 (2001): 48–56. 11488131

[27] M. V. Korolenkova , A. A. Poberezhnaya , and L. Andreyashkina , “Dental Age in Children With Epidermolysis Bullosa,” Stomatologiya 100, no. 4 (2021): 68. 10.17116/stomat202110004168 34357731

[28] H. Shah , F. McDonald , V. Lucas , P. Ashley , and G. Roberts , “A Cephalometric Analysis of Patients With Recessive Dystrophic Epidermolysis Bullosa,” Angle Orthodontist 72, no. 1 (2002): 55–60. 11843275 10.1043/0003-3219(2002)072<0055:ACAOPW>2.0.CO;2

[29] J. T. Wright , S. Cashion , and R. Hoover , “The Esthetic Stainless Steel Crown Bridge: Report of Two Cases,” Pediatric Dentistry 21, no. 2 (1999): 137–141. 10197344

[30] J. K. Brooks , L. C. Bare , J. Davidson , L. S. Taylor , and J. T. Wright , “Junctional Epidermolysis Bullosa Associated With Hypoplastic Enamel and Pervasive Failure of Tooth Eruption: Oral Rehabilitation With Use of an Overdenture,” Oral Surgery, Oral Medicine, Oral Pathology and Oral Radiology 105, no. 4 (2008): e24–e28. 10.1016/j.tripleo.2007.12.038 18329564

[31] N. K. O. Fonseca , V. D. Curtarelli , J. Bertoletti , et al., “Avoidant Restrictive Food Intake Disorder: Recent Advances in Neurobiology and Treatment,” Journal of Eating Disorders 12, no. 1 (2024): 74. 38849953 10.1186/s40337-024-01021-z PMC11157884

[32] A. B. Tanner and T. K. Richmond , “Assessing Growth in Children and Adolescents With Avoidant/Restrictive Food Intake Disorder,” Journal of Eating Disorders 12, no. 1 (2024): 82. 38877582 10.1186/s40337-024-01034-8 PMC11177361

[33] J. Green , “Clinical Orthodontic Care for Patients With Epidermolysis Bullosa,” Dental Nurse 8, no. 6 (2012): 1–6.

[34] C. Arriagada‐Vargas , M. T. Abeleira‐Pazos , M. Outumuro‐Rial , et al., “Rare Disorders: Diagnosis and Therapeutic Planning for Patients Seeking Orthodontic Treatment,” Journal of Clinical Medicine 11, no. 6 (2022): 1527. 35329854 10.3390/jcm11061527 PMC8954363

[35] A. C. Mühlbacher and C. Juhnke , “Patient Preferences Versus Physicians″ Judgement: Does It Make a Difference in Healthcare Decision Making?” Applied Health Economics and Health Policy 11, no. 3 (2013 ): 163–180. 23529716 10.1007/s40258-013-0023-3

