# Supplementary material for: Orthodontic Treatment in Patients With Epidermolysis Bullosa (EB)—Clinical Practice Guidelines (CPG)
Source: Spec Care Dentist. 2025 Sep 3;45(5):e70084. doi: 10.1111/scd.70084 (PMC12406292; doi:10.1111/scd.70084)
Supplement: Supplementary file 1 — Supplementary Table 1: List of participants Supplementary Table 2: List of Recommendations and agreement Round 1 Supplementary Table 3: List of Recommendations and agreement Round 2 Supplementary Table 4: Patients and non‐dentist healthcare professionals perspectives [file SCD-45-0-s001.docx]

**SUPPLEMENTARY INFORMATION**

**Supplementary Table 1: List of participants**

[BLINDED]

**Supplementary Table 2: List of Recommendations and agreement Round 1.**

| **Supplementary Table 1. List of Recommendations and agreement Round 1.** | | | | |
| --- | --- | --- | --- | --- |
| **SECTION** | **QUESTION** | **RECOMMENDATION** | **I AGREE** | **I DISAGREE** |
| **Section 1. General information about dentofacial orthopaedic and orthodontic treatment in Epidermolysis Bullosa (EB).** | **1.1 Is orthodontic and/or dentofacial orthopaedic (DO) treatment possible in patients living with EB?** | Dentofacial orthopaedic and/or orthodontic treatment is possible in patients living with EB. | 100% | 0% |
|  |  | Best Practice (BP): Proper risk-benefit assessment should be performed before treatment to identify variables related and not related to EB that can affect orthodontic treatment. | 100% | 0% |
|  | **1.2 Is orthodontic and/or DO treatment the same in complexity for all patients with EB?** | Orthodontic and/or dentofacial orthopaedic treatment is not the same in complexity for all patients with EB. | 100% | 0% |
|  | **1.3 At what age should patients diagnosed with EB be referred to an orthodontist for their initial orthodontic evaluation?** | Patients with EB should be referred for an orthodontic evaluation at the age of 7 or as soon as any orthodontic problems are detected. | 90.9% | 9.10% |
| **Section 2. Orthodontic and DO diagnosis in Epidermolysis Bullosa (EB).** | **2.1 What main characteristics can be observed in patients with EB?** | Several clinical characteristics affect patients with EB including extraoral and intraoral features, oral functions, and systemic conditions, especially in high-risk EB types, such as RDEB, JEB, and KEB. | 100% | 0% |
|  |  | Best Practice (BP): IIndividual assessment should be performed to identify all extraoral and intraoral features, and oral functioning characteristics that might have an impact on the diagnosis and treatment plan. | 90.9% | 9.10% |
|  | **2.2 What types of malocclusions or orthodontic problems are most common among EB patients?** | The most common malocclusions described in EB are teeth crowding and posterior crossbite. Specific subtypes of EB can present distinctive features, such as reduced maxillary size in RDEB and failure of teeth eruption in JEB. | 81.8% | 18.20% |
|  | **2.3Which methods are necessary to achieve a successful dentofacial orthodontic diagnosis in patients with EB?** | General management: Patients with EB require specific skin and mucosal management, which includes the utilisation of lubrication in lips, careful use of suction and air-water syringes, employing a controlled local anaesthesia technique, and oral bullae drainage. Additional padding of the dental chair may aid patients with severe RDEB in ensuring their comfort during treatment. Clinical facilities must be accessible to patients using wheelchairs (Figure 2, A). If the facilities or team are not trained to treat patients with EB, educating them and creating awareness about the specific management protocols for these patients is advisable. For general management of patients with EB, refer to the Clinical Practice Guidelines: Oral health care for children and adults living with epidermolysis bullosa; chapter 3: Oral health care and dental treatment for children and adults living with epidermolysis bullosa—Clinical practice guidelines[(3)](https://www.zotero.org/google-docs/?M3wrLH). | 100% | 0% |
|  |  | Facial examination: Dressings, frequent in areas such as the neck, ear, and eyes, might limit clinical examination (Figure 2, B and C). | 90.9% | 9.10% |
|  |  | Intraoral examination: Patients with EB of all types may present with open wounds or lesions on their lips, commissures, and oral cavity. The degree of mucosal fragility may range from extreme fragility to normal strength. In patients with RDEB, an appropriate intraoral examination can be severely impaired by oral strictures (microstomia, ankyloglossia, and vestibule obliteration). Measuring teeth crowding intraorally might not be possible. Patients diagnosed with JEB may exhibit areas of chronic granulation tissue, both periorally and intraorally. Patients with other subtypes of EB may also face limitations according to their phenotype expression. Patients who have a history of tube feeding may experience oral-sensory difficulties. In these patients, a multidisciplinary approach is advised with speech and language therapists. Functional analysis: might be limited due to strictures, lesions and pain. | 100% | 0% |
|  |  | Records assessment.Extraoral photographs: Patients with considerable facial lesions may be reluctant to take extraoral photographs. Intraoral photographs: Normal intraoral photographs with occlusal intraoral mirrors, contrasters, and lip retractors might be impossible to use without causing damage to the patient. Hence, in patients with severe contractures or fragility, only smile pictures might be possible to take. Since some patients have photophobia and a high visual sensitivity, flash should be used with caution. | 100% | 0% |
|  |  | Dental casts: Dental trays and intraoral scanning are difficult to fit without causing harm, mostly due to reduced mouth opening and the lack of mobility of the soft tissues. If necessary, it is recommended to use customised or flexible standard trays[(7)](https://www.zotero.org/google-docs/?6R3bzk). Intraoral scanner use may be limited to the anterior zone primarily because of the size of the camera and the movement required for its use. | 90.9% | 9.10% |
|  |  | Radiographs: Radiographs: Extraoral X-rays, such as panoramic, cephalometric, or cone beams, are recommended for orthodontic treatment and do not necessitate any significant modifications. In patients with JEB, the teams must establish proper radiographic follow-up to early identify signs of crown resorption and tooth retention. Intraoral X-rays, if needed, may be difficult to obtain due to ankyloglossia and mucosal fragility. | 90.9% | 9.10% |
|  |  | Other specific complementary exams: If dental casts are needed and it is not possible to obtain impressions or scanners, stereolithographic models can be used. | 90.9% | 9.10% |
| **Section 3. Orthodontic and DO treatment in Epidermolysis Bullosa (EB).** | **3.1 What are the benefits of orthodontic treatment in patients with EB?** | Orthodontic treatment has the potential to benefit patients with EB. The reported benefits include enhanced aesthetics, easier oral hygiene, and fewer traumatic ulcers in well aligned teeth. Before orthodontic treatment, a proper risk-benefit assessment is mandatory. During orthodontic treatment, patients can present transitory pain, wounds, and an increased risk of caries due to difficulties performing oral hygiene with fixed appliances. | 100% | 0% |
|  | **3.2 What are the multidisciplinary areas that patients with EB may require prior to, during, or subsequent to receiving orthodontic treatment?** | Orthodontic treatment requires a multidisciplinary approach, including special care dentistry, paediatric dentistry, oral rehabilitation, periodontology and speech therapy. Additional specialities that may need to be part of the team can be, but are not limited to: maxillofacial surgery, dermatology, paediatrics, nutrition and psychology. | 100% | 0% |
|  | **3.3 Which considerations should be taken into account when planning orthodontic treatment for patients with EB?** | Orthodontic planning for patients with EB must be realistic and patient-centred. | 100% | 0% |
|  |  | The treatment objectives must be realistic in light of the severity of the malocclusion and adapted to the orofacial and psychosocial implications of EB. Common treatment objectives include achieving tooth alignment, improving aesthetic complaints, enhancing transverse maxillary dimension, and providing occlusal stability[(8,18)](https://www.zotero.org/google-docs/?pQypzi). | 90.9% | 9.10% |
|  |  | Treatment should be planned in stages, continuing to the next stage once the objective has been achieved. | 100% | 0% |
|  |  | The duration of treatment in RDEB patients is significantly longer, both during each session in the dental chair and throughout the entire treatment process, compared to other EB and non-EB patients. | 100% | 0% |
|  |  | Therapeutic alliance and behavioural support are important and cannot be overstated, as orthodontic treatment can extend for several years. Behavioural support techniques can include pharmacological techniques (sedation) for some specific clinical procedures, such as long sessions for bracket bonding or mini-implant positioning. Still, they are not recommended for routine treatment sessions, such as regular orthodontic visits[(3)](https://www.zotero.org/google-docs/?7UdCV9). | 100% | 0% |
|  |  | In some cases, the patient or the clinical team may decide to interrupt or stop treatment. Patients may wish to discontinue the treatment due to discomfort or ulcers associated with the appliances. Clinicians may indicate to pause or stop the treatment because of poor oral hygiene with an increased number of caries, or when the patient presents additional systemic complications that make it difficult to attend the routine monthly session. In these cases, all orthodontic appliances should be removed and, if possible, the occlusion should be stabilised with aligners or thermoplastic devices. | 100% | 0% |
|  | **3.4 Which adjustments can be implemented to adapt orthodontic techniques for patients with EB?** | Orthodontic treatment may benefit from specific adjustments depending on the phenotypic expression of each subtype of EB, especially in RDEB, KEB and JEB:  Early/selective teeth extraction: As patients with severe RDEB present reduced alveolar arches leading to severe crowding, it has been suggested to perform early/selective teeth extractions to guide the eruption process into a better-aligned arch. Reported experiences have successfully improved teeth crowding and oral hygiene after the extraction of the first premolars. Uncommon extraction patterns have also been reported, such as a patient with severe microstomia (mouth opening of 6mm) in whom both upper permanent canines were extracted due to severe lack of space, limited access to continuous dental treatment, and already well-aligned lateral incisors and premolars[(13,14)](https://www.zotero.org/google-docs/?Xbq3L7). | 90.9% | 9.10% |
|  |  | Removable dentofacial orthopaedic appliances: Taking an impression can be challenging in patients with severe microstomia. In question 2.3, section 2b of the present document, different methods for taking impressions, such as custom-made or flexible trays, intraoral scanners and stereolithography, are described[(7)](https://www.zotero.org/google-docs/?HeN2ec). Modifications of the appliance include covering the retentive structures with acrylic, which can reduce the risk of wounds due to metallic structures such as clasps and springs[(33)](https://www.zotero.org/google-docs/?bAEQmY). In the case of removable appliances, these must be tooth-worn, as the pressure and pain associated with mucosal support might reduce compliance with the therapy[(3)](https://www.zotero.org/google-docs/?o7qSkG). | 100% | 0% |
|  |  | Fixed dentofacial orthopaedic appliances: Fixed palatal expanders, such as Hyrax, can be used in EB. Metallic bands, bonded acrylic expanders, or mini–implants can be used for anchorage. One of the limitations of the technique is the difficulty in obtaining impressions and adapting the bands. There is not enough evidence to support one anchorage technique over the other. To select the proper technique it is important to consider the potential discomfort associated with the expansion protocol and the possibility of ulcers arising from the interaction between the Hyrax and the mucosa. Ideally, mucosal support should be avoided[(3,8,13)](https://www.zotero.org/google-docs/?LMBSTr) (Figure 6, A). Bite ramps are also used to manage occlusion during mixed dentition, especially in the anterior teeth. | 100% | 0% |
|  |  | Fixed braces: Metallic fixed braces, whether regular or self-ligating, are a treatment alternative in patients with different subtypes of EB, including RDEB. Characteristics such as microstomia, vestibule obliteration, ankyloglossia, and mucosa fragility may make bonding technique difficult, especially in the posterior area. As wire changes can cause ulcers and pain; some authors have suggested limiting the orthodontic treatment to the anterior teeth[(18)](https://www.zotero.org/google-docs/?iOd21t). If brackets are planned on the anterior teeth only, orthodontic anchorage on the posterior area will be restricted, making it challenging to perform retrusive movements. In other words, tooth extraction to retrude anterior teeth should only be performed if posterior anchorage has been successfully secured[(18)](https://www.zotero.org/google-docs/?Xakuie). Other examples of adjustments to facilitate treatment include performing a vestibuloplasty prior to treatment in patients with severe contractures[(7)](https://www.zotero.org/google-docs/?KgWl4D) or removing the hook from the brackets/bands[(3)](https://www.zotero.org/google-docs/?lKpR0l). Patients with JEB and AI may also encounter bonding difficulties owing to the altered enamel structure or the restored teeth (metallic, polycarbonate, acrylic and ceramic crowns, composite, and glass Ionomer). In cases of hypoplastic AI, the bonding technique might require adjustments. An example reported in the literature is the use of 5% sodium hypochlorite as an etching agent and All-Surface bonding agents (Assure-Plus by Reliance)[(8)](https://www.zotero.org/google-docs/?pqp9HQ). | 100% | 0% |
|  |  | Mini-implants: Successful use of mini-implants has been reported in RDEB and JEB patients. They were used to reinforce the anchorage with fixed braces and as anchorage to perform maxillary rapid palate expansion (MARPE). The protocols used were similar to the general population, however, the patient with RDEB required surgery to increase mouth opening and vestibule depth before placing the mini-implants[(8,18)](https://www.zotero.org/google-docs/?nFb2xC). | 81.8% | 18.20% |
|  |  | Aligners: Successful use of aligners has been reported in RDEB. Nevertheless, adjustments were necessary to obtain the impression, as microstomia limited intraoral scanning. An impression made using adhesion elastomeric silicone on a flexible tray allowed to obtain a cast, additionally, digital improvement of silicone defects was conducted prior to printing the aligners[(7)](https://www.zotero.org/google-docs/?in0A96). When planning aligner therapy, it is important to design the treatment trying to consider attachments in the anterior area mainly, as bonding is difficult and challenging in the posterior region of those patients with oral contractures. The risk of caries should be considered when stripping in RDEB | 100% | 0% |
|  | **3.5 Is orthodontic retention necessary for individuals with EB?** | Orthodontic retention is highly recommended, especially for patients with a functional imbalance or a high risk of malocclusion relapse. | 100% | 0% |
|  |  | BP: The retention types should be selected according to the clinical features, feasibility and risk of caries. | 100% | 0% |
|  | **3.6 What are the complications associated with orthodontic treatment in individuals diagnosed with EB?** | Complications associated with orthodontic treatment in individuals diagnosed with EB may include, but are not limited to, oral ulcers, pain, poor oral hygiene with an increased number of caries, and recurrent debonding of braces. | 100% | 0% |
|  | **3.7 What considerations are necessary for maintaining oral hygiene in patients with EB who are undergoing orthodontic treatment?** | Oral hygiene of patients with EB undergoing orthodontic treatment is more complex and requires precise guidance for both the patients and/or their carers. | 90.9% | 9.10% |
|  |  | The brushing technique should be complemented with specific hygiene elements and products, which should be monitored by the guardian. | 90.9% | 9.10% |
|  |  | Frequent professional hygiene and preventive measures, such as fluoride varnish, must be carried out by the patient's general dentist. | 81.8% | 18.20% |
|  | **3.8 What are the barriers patients with EB face when it comes to orthodontic treatment?** | Patients with EB who need orthodontic treatment can face barriers due to their EB, such as pain, wound, anxiety, and other associated systemic complications; and barriers in their social environment, such as funding and availability of specialised services locally. From a professional perspective, the lack of training in rare diseases of orthodontists can be the main barrier. To overcome these barriers, nationally funded reference centres could be an aid in improving access to orthodontics for patients with EB. Local barriers must be identified and addressed. | 100% | 0% |
|  | **3.9 What are the adjustments for orthodontic treatment needed by patients with EB subtypes with low and moderate risk of oral and dental manifestations and complications?** | Patients with EB with low and moderate risk of oral manifestations will need adjustments according to their individual phenotypic expression, considering oral blisters and ulcers. In general, protocols for non-EB patients can be applied. | 100% | 0% |
| **FIGURES** | **Question 2.1. What characteristics can be observed in patients with EB seeking orthodontic treatment?** | Figure 1: Characteristics of patients with EB subtypes with high risk of oral and dental manifestations and complications. A) Generalised hypoplastic Amelogenesis Imperfecta in an intermediate JEB patient. B) Several caries and anterior crossbite in #11 in a patient with severe RDEB. C) Chronic intraoral granulation tissue lesion in a severe JEB patient. D) Perioral and intraoral granulation tissue in a patient with severe JEB. E) Ankyloglossia and microstomia in a patient with severe RDEB. F) Pseudosyndactyly in a patient with severe RDEB. | 90.9% | 9.10% |
|  | **Question 2.3 Which methods are necessary to achieve a successful dentofacial orthodontic diagnosis in patients with EB?** | Figure 2: A) A patient with RDEB, a wheelchair user receives treatment in a wheelchair recliner. B) An extraoral photograph of a patient with an eye ulcer covered by a dressing. C) An extraoral photograph of a dressing covering chronic lesions on the neck and ear. D and E) A custom-designed acrylic lower tray was used for a patient with RDEB, but despite the efforts, a hemorrhagic lesion was produced on the right commissure. | 90.9% | 9.10% |
|  | **Question 3.1 What are the benefits of orthodontic treatment in patients with EB?** | Figure 3: Orthodontic treatment in a patient with intermediate RDEB. A) Severe malocclusion with an anterior crossbite in #12 and #22, both with caries associated with difficulties in performing oral hygiene due to malposition. B) and C) Evolution of orthodontic treatment after 5 and 10 months, respectively. Treatment planning considered only upper arch brackets, realistic objectives (Improving upper alignment for better aesthetic and easier oral hygiene) and orthodontic strategies (Difficulty in bonding posterior tubes and brackets, meaning limited anchorage and future difficulties in space closing. Hence, the team decided not to perform first premolars extractions). D) One year after treatment, the alignment of anterior teeth was successful, and lateral incisors have been restored. | 90.9% | 9.10% |
|  | **Question 3.2 What are the multidisciplinary areas that patients with EB may require prior to, during, or subsequent to receiving orthodontic treatment?** | Figure 4: Strictures release surgery before taking impressions for the aligners. A) Vestibuloplasty B) Mucosal strictures release surgery to increase mouth opening. | 81.8% | 18.20% |
|  | **Question 3.3 Which considerations should be taken into account when planning orthodontic treatment for patients with EB?** | Figure 5: A) Male patient with intermediate RDEB at the age of 5 years presented anterior and posterior crossbite. B) Maximal mouth opening 20 mm. C) Early intervention was planned. Dental casts were obtained by using a custom-made acrylic tray. A removable expansion appliance was conceived. The patient did not like using the appliance and never wore it. The treatment was suspended because of a lack of compliance. D) The family made a request for a second treatment attempt at the age of 8. This time, a fixed appliance was planned, designed and bonded. E) The patient refused to eat with the appliance in mouth. A week after bonding the appliance had to be removed. Treatment was stopped for the second time. | 81.8% | 18.20% |
|  | **Question 3.4 Which adjustments can be implemented to adapt orthodontic techniques for patients with EB?** | Figure 6: A) A removable dentofacial orthopaedic appliance in a patient with localised DDEB. B) Fixed metallic brackets in a patient with severe RDEB. C) A patient with intermediate JEB with four mini-implants placed on the palate for bonding a MARPE appliance (Mini-implant assisted rapid palate expansion). On the occlusal surfaces a temporary retention device is used. D) Aligners in a patient with intermediate RDEB. | 81.8% | 18.20% |
|  | **3.9 What are the adjustments for orthodontic treatment needed by patients with EB subtypes with low and moderate risk of oral and dental manifestations and complications?** | Figure 7: Patient with moderate risk of oral manifestations (DDEB) during (A) Dentofacial orthopaedic and (B) orthodontic treatment. The treatment followed the protocols used for non-EB patients. The patient occasionally utilised orthodontic wax. | 90.9% | 9.10% |

**Supplementary Table 3: List of Recommendations and agreement Round 2.**

| **SECTION** | **QUESTION** | **RECOMMENDATION** | **I AGREE** | **I DISAGREE** |
| --- | --- | --- | --- | --- |
| **Section 2. Orthodontic and DO diagnosis in Epidermolysis Bullosa (EB).** | **2.3 Which methods are necessary to achieve a successful dentofacial orthodontic diagnosis in patients with EB?** | Dental casts: Dental trays and intraoral scanning are difficult to fit without causing harm, mostly due to reduced mouth opening and the lack of mobility of the soft tissues. If necessary, it is recommended to use customised or flexible standard trays[(7)](https://www.zotero.org/google-docs/?V2tlqA). Intraoral scanner use may be limited to the anterior zone primarily because of the size of the camera and the movement required for its use. **Some participants, although they agreed with the recommendation, suggested that it is possible to take an impression of the anterior zone with silicone.** | 90.9% | 9.10% |
|  |  | **New recommendation** = Dental casts: In patients with severe microstomia, dental trays and intraoral scanning are difficult to fit without causing harm, mostly due to reduced mouth opening and the lack of mobility of the soft tissues (Figure 2, D and E). If necessary, it is recommended to use customised or flexible standard trays[(7)](https://www.zotero.org/google-docs/?b4MbTD). Impressions with silicone can be used to obtain models from the anterior zone of the arch. In some cases, an intraoral scanner can be used only in the anterior zone, primarily because of the size of the camera and the movement required for its use. |  |  |
|  |  | Radiographs: Radiographs: Extraoral X-rays, such as panoramic, cephalometric, or cone beams, are recommended for orthodontic treatment and do not necessitate any significant modifications. In patients with JEB, the teams must establish proper radiographic follow-up to early identify signs of crown resorption and tooth retention. Intraoral X-rays, if needed, may be difficult to obtain due to ankyloglossia and mucosal fragility. **Some participants suggested incorporating that the procedure must be carried out or assisted by an individual with knowledge of EB.** | 81.8% | 18.20% |
|  |  | **New recommendation=** Radiographs: Extraoral radiographs, such as panoramic, cephalometric, or cone beams, are recommended for orthodontic treatment and do not necessitate any significant modifications. In patients with JEB, the teams must establish proper radiographic follow-up to early identify signs of crown resorption and tooth retention. However, patient positioning should be carried out or assisted by an individual with knowledge of EB. |  |  |
| **Section 3. Orthodontic and DO treatment in Epidermolysis Bullosa (EB).** | **3.7 What considerations are necessary for maintaining oral hygiene in patients with EB who are undergoing orthodontic treatment?** | The brushing technique should be complemented with specific hygiene elements and products, which should be monitored by the guardian. **Some participants addressed that not all patients under orthodontic treatment are children. Therefore, recommendations should be suitable for patients with and without a guardian.** | 100% | 0% |
|  |  | **New recommendation**= The brushing technique should be complemented with specific hygiene elements and products. In children and adults with limited manual dexterity, it is advisable that the carers assist the brushing technique. |  |  |
| **FIGURES** | **Question 2.1. What main characteristics can be observed in patients with EB?** | Figure 1: Characteristics of patients with EB subtypes with high risk of oral and dental manifestations and complications. A) Generalised hypoplastic Amelogenesis Imperfecta in an intermediate JEB patient. B) Several caries and anterior crossbite in #11 in a patient with severe RDEB. C) Chronic intraoral granulation tissue lesion in a severe JEB patient. D) Perioral and intraoral granulation tissue in a patient with severe JEB. E) Ankyloglossia and microstomia in a patient with severe RDEB. F) Pseudosyndactyly in a patient with severe RDEB. **It was suggested that picture B was not clear enough (B: Several caries and anterior crossbite in #11 in a patient with severe RDEB)** | 90.9% | 9.10% |
|  |  | **New recommendation:** New picture B and new text: B) Severe crowding and microstomia in a patient with severe RDEB. |  |  |

**Supplementary Table 4: Patients and non-dentist healthcare professionals' perspectives.**

| **Patients' perspective** |
| --- |
| [BLINDED], , 28 years old, severe RDEB: “*Orthodontic treatment was fundamental for improving my Quality of Life and oral function. Due to my severe condition, I never thought I could receive orthodontic treatment, but luckily, the dental team was able to provide it. I was treated with aligners due to the lack of space for my tongue, my lower incisors were retroclined causing me wounds and problems for eating. Orthodontic treatment changed this and currently, I have fewer wounds, can open my mouth bigger and brush my teeth better, improving my QoL. I wish there were more procedures for people with severe types of EB, like me, because I have a small mouth and obtaining casts of my mouth was highly complex. Access to orthodontic treatment is still very limited to people with RDEB, as some patients still cannot access proper dental treatment, or the professionals do not feel confident enough to treat us. Therefore, it is important to provide early access to preventive dental programs for patients with EB, including orthodontics. This CPG may help more patients to access, in the future, orthodontic treatment as I did”.* |
| [BLINDED], , 26 years old, Intermediate RDEB: *"My experience with orthodontic treatment wasn’t bad, but I was very uncomfortable with having them on, especially when eating. Eating was really difficult for me and my gums hurt a lot. I lasted about two months and decided to take them off. Also, for aesthetic reasons and quality of life, it would be good to have a proper bite, but considering how long the treatment was going to take, it was a long process. In two months, I lost about 4 kg because I couldn’t eat or it was hard for me to eat things like bread or meat. Not being able to eat was the main reason I decided to take the braces off."* |
| **Non-dentist healthcare professionals' perspectives.** |
| [BLINDED], , Paediatric dermatologist, EB expert: *Primary care providers and dermatologists, especially paediatric dermatologists, who care for individuals with EB should be aware that orthodontic treatment is possible and often necessary for their patients. Ideally, they should have a good referral relationship to a dentist, especially a paediatric dentist for the appropriate age group. With these guidelines, primary care providers should be better able to help dentists to collaborate with orthodontists who are willing to treat patients with EB. It appears that many primary care providers and dentists believe that it is not possible to perform orthodontic/orthopaedic procedures on this population, and these guidelines will be very helpful. However, not all EB patients are good candidates for orthodontics, and this guideline is helpful in emphasizing the importance of pre-treatment discussion and evaluation to determine the goals of the patient and limitations of each situation. The several international locations of the authors will be extremely helpful in guiding providers to experienced orthodontic practitioners close to their patients homes and/or helping local orthodontists to provide the best possible care.* |
| [BLINDED], , Dermatologist, EB expert: “*EB is a complex disease that highlights the essential role of the skin and mucosal membranes as barriers between body and environment. The oral cavity is a critical organ for nutrition and other physiological and social functions. It is impressive and dramatic to see young children with EB not being able to eat because of oral strictures, painful ulcerations, and teeth, and the reluctance of many dentists to apply appropriate treatments. This is mainly due to a lack of expertise and confidence, and to long treatment times and costs. This CPG fills the gap due to limited literature, which is because most dentists treat a small number of patients but also because of the spiral of novelty imposed by scientific journals that hardly publish case reports or small case series.*  *Therefore, expert opinion and recommendations are precious and provide a high evidence level. The economic aspect is more difficult to solve, particularly in countries that do not financially support EB centers or the special care this small but vulnerable group of patients need. Nevertheless, this practical guideline can provide an argument and basis in the battle between the patients/physicians and the payer.”* |
| [BLINDED], , Psychologist, EB Expert: "*Orthodontic care for patients with epidermolysis bullosa (EB) can be very challenging due to the fragility of oral tissues, heightened pain sensitivity, and limited mouth opening. In addition, facial and dental esthetics contributes to self-esteem, especially in younger patients. For this reason, treatment protocols should be personalized and prioritized towards non-irritating appliances and experiences. Orthodontic treatment should be delivered with consideration of esthetics, but never at the expense of the patient's comfort. From a psychological perspective, being sensitive to anxiety and building trust is a priority as patients may associate dental care with pain and trauma. Providing a compassionate, patient-centered approach, clear communication, and psychological support can help alleviate fears and improve treatment adherence, ultimately enhancing both oral health and overall well-being. A multidisciplinary approach, as teamwork among dermatologists, pain specialists, and psychologists, to support care ensures addressing both physical and emotional needs throughout the entire orthodontic process".* |
| [BLINDED], Speech and Language Therapist (SLT)*: "EB can have serious repercussions on orofacial function, including disruptions to vital functions such as breathing and swallowing. This also includes alterations in chewing, speech, and facial expression. While not vital, these functions are characterized by the high motor specificity required for their execution. The skin and mucosal changes that characterize EB in its intermediate and severe stages can have a significant impact on these functions and require in-depth, specialized analysis. Therefore, interdisciplinary collaboration between orthodontists and SLTs is essential, as managing orofacial functions from the start of orthodontic treatment improves the long-term prognosis. It is important for dentists to recognize the importance of orofacial functions and consider a personalized and interdisciplinary therapeutic approach in this area, as this can be crucial for achieving an adequate quality of life in everyday life, minimizing the functional limitations associated with the condition".* |
